# Structure-Based Design of Inhibitors of the m^6^A-RNA Writer Enzyme METTL3

**DOI:** 10.1021/acsbiomedchemau.3c00023

**Published:** 2023-06-14

**Authors:** Rajiv Kumar Bedi, Danzhi Huang, Yaozong Li, Amedeo Caflisch

**Affiliations:** Department of Biochemistry, University of Zurich, Winterthurerstrasse 190, Zurich CH-8057, Switzerland

**Keywords:** METTL3/METTL14, epitranscriptomics, computer-aided drug design (CADD), molecular dynamics, m^6^A-RNA, SAR

## Abstract

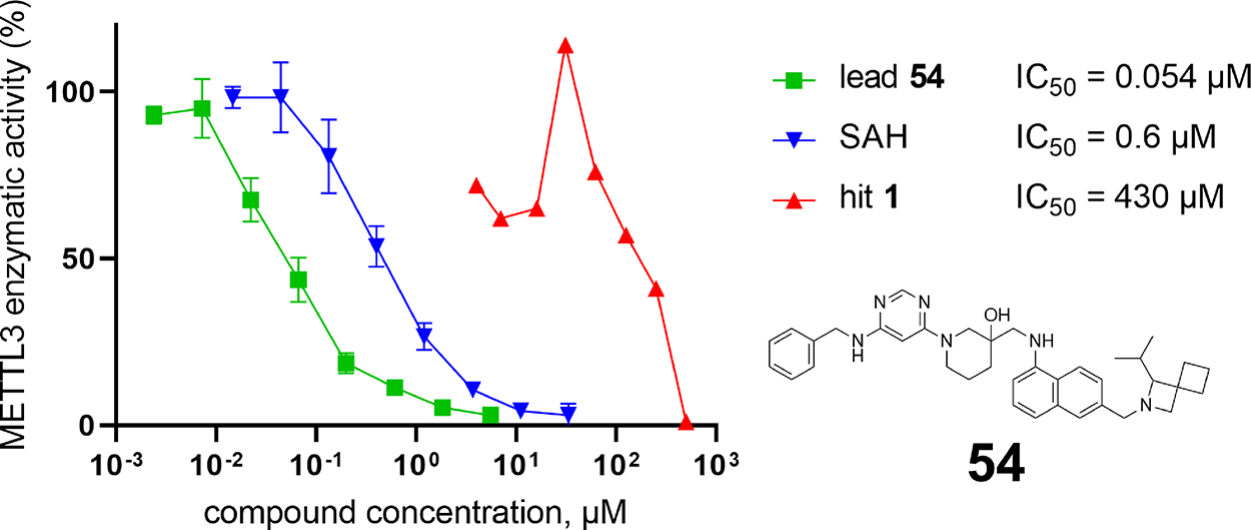

Methyltransferase-like 3 (METTL3) and METTL14 form a heterodimeric complex that catalyzes the most abundant internal mRNA modification, *N*^6^-methyladenosine (m^6^A). METTL3 is the catalytic subunit that binds the co-substrate *S*-adenosyl methionine (SAM), while METTL14 is involved in mRNA binding. The m^6^A modification provides post-transcriptional level control over gene expression as it affects almost all stages of the mRNA life cycle, including splicing, nuclear export, translation, and decay. There is increasing evidence for an oncogenic role of METTL3 in acute myeloid leukemia. Here, we use structural and dynamic details of the catalytic subunit METTL3 for developing small-molecule inhibitors that compete with SAM. Starting from a hit identified by high-throughput docking, protein crystallography and molecular dynamics simulations were employed to guide the optimization of inhibitory activity. The potency was successfully improved by 8000-fold as measured by a homogeneous time-resolved fluorescence assay. The optimized compound is selective against the off-targets RNA methyltransferases METTL1 and METTL16.

## Introduction

RNA modifications have evolved as a survival tool developed by nature to alter gene expression at the post-transcriptional level.^1^*N*^6^-methyladenosine (m^6^A) is the most abundant RNA modification and is preferentially enriched within 3′ UTRs and around stop codons. In mammals, over 18,000 transcripts of more than 7000 genes within a consensus sequence of DRACH (D = A/G/U; R = A/G; H = A/C/U) have m^6^A modification.^2^ The m^6^A modification exists on mRNA, tRNA, rRNA, small nuclear RNA (snRNA), and several long noncoding RNA, such as *Xist*.^3^ The m^6^A modification on the mRNA transcript leads to its structural changes, which regulate downstream events controlling almost every aspect of cell proliferation. As a result, m^6^A associates with various physiological processes, and the scientific community is finding the ever-growing links between m^6^A and many human diseases. The link between m^6^A and various cancer types has also been reported, including leukemia, sarcoma, mesothelioma, stomach cancer, prostate cancer, breast cancer, pancreatic cancer, and kidney cancer.^4^ Recent data shows that a decrease in the m^6^A level causes apoptosis and reduces the invasiveness of cancer cells.^5^ The m^6^A modification of mRNA is regulated to homeostasis by its methyltransferase (writer), demethylase (eraser), and recognition (reader) proteins.^6^ METTL3/METTL14 forms the writer complex in which METTL3 is the catalytic subunit, and METTL14 stabilizes the heterodimer interface and facilitates the substrate mRNA recognition and binding. Thus, the METTL3/METTL14 complex is an attractive target for inhibition by small molecules (for comprehensive and detailed reviews on METTL3 inhibitor development, see refs (7, 8)), but to date, only two series of potent and selective inhibitors have been published.^9,10^

In this work, we study the catalytic subunit of the METTL3/METTL14 writer complex in atomic and dynamic details using X-ray crystallography and molecular dynamics (MD) simulations. Starting with a hit compound with a high μM affinity obtained by docking, we optimized its scaffold by performing several rounds of modifications. With each modification, we learned about critical interactions of the inhibitors with the protein and we made use of crystal structures for improving the affinity. The MD simulations revealed how the catalytic site behaves in its apo form. Our simulations also capture the plasticity of three loops that surround the catalytic site. The intrinsic flexibility of part of the SAM-binding pocket of METTL3 provides unique opportunities for developing selective inhibitors.

## Results and Discussion

### Structural Analysis and Molecular Dynamics Reveal an Aromatic Pocket for Improving Binding Affinity

**Figure 1:**
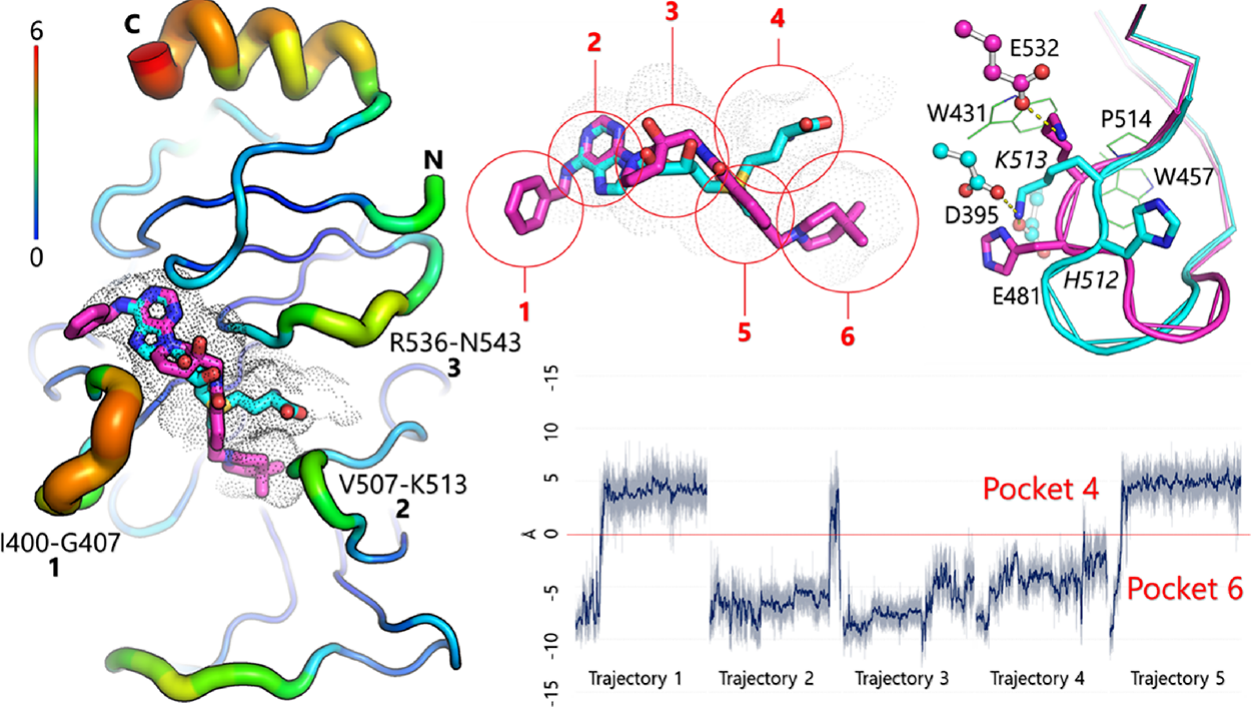
SAM binding site flexibility and aromatic cage for the hit-to-lead optimization. Left: Crystal structures of SAM (carbon atoms in cyan, PDB ID: 5L6E) and UZH1a (compound **43**, magenta, PDB ID: 7ACD) in complex with METTL3. The three loops are labeled by their number and the residue range. The tube size and color reflect the backbone root-mean-square fluctuations (RMSFs) calculated from one of the 0.5 μs MD runs. The accessible space of the SAM binding site is also shown (black dots). Top middle: SAM and UZH1a binding poses with numbering of the individual pockets of the SAM binding site. Top right: Castling like switching of the orientations of the H512 and K513 side chains. The three residues of the aromatic cage are shown (W431, W457, and P514; green). Bottom right: Time series of K513 orientation. The side chain of K513 switches from pocket 6 to pocket 4 in three of the five 0.5 μs MD runs of apo METTL3. The distance *d* = *d*_1_–*d*_2_ (*d*_1_, K513 (NZ) to E481 (CD); *d*_2_, K513 (NZ) to E532 (CD)) is used to monitor the K513 switching. The MD simulations were carried out by CHARMM and NAMD.^15,16^

METTL3 and METTL14 form the heterodimer functioning as the m^6^A writer. The METTL3 subunit serves as the catalytic domain, and METTL14 plays an allosteric role in stabilizing the complex and the substrate mRNA binding. The catalytic site of METTL3 is wrapped by three flexible loops: loop 1 (I400–G407), loop 2 (V507–K513), and loop 3 (R536–N543).^11−13^ These loops might regulate cofactor and substrate binding and/or release of the products. Crystal structures (apo and holo) and our MD simulations show that loop 2 can adopt two different conformations and is stabilized by multiple salt bridges. In pocket 6, D395 and E481 form a salt bridge with K513, while in pocket 4, K513 forms a salt bridge with E532 (Figure 1). Upon the SAM binding, K513 is pushed out of pocket 4 by SAM’s zwitterionic tail, and K513 interacts with D395 and E481 instead. Meanwhile, H512 is disconnected from D395 and flips out of the catalytic site, thus unblocking pocket 6.

To study the intrinsic flexibility of the catalytic site, we conducted five independent MD simulations (collectively 2.5 μs) by starting with the apo conformation of METTL3/METTL14 (see Materials and Methods). We find that the conformations of loop 2 are interchangeable in the apo protein, which suggests that either of the two conformations might be exploited for the design of small-molecule inhibitors (Figure 1). The side chain of K513 switches between E532, D395, and E481 and thereby reshapes and stabilizes the catalytic site in different conformations. When K513 is sandwiched between D395 and E481, it blocks pocket 6, similar to the SAM/SAH-bound structures (PDB ID: 5IL1 and 5IL2).^14^ By contrast, K513 can alternately reside in pocket 4, and this conformational change allows the opening of pocket 6. The plot of a distance that monitors the orientation of K513 (d) and its population shows that pocket 4 and pocket 6 are almost equally accessible (Figure S1).

Utilizing docking campaigns as in our previous work,^17^ we identified the hit compound **1** (Table 1). Its binding mode was confirmed by crystallography in complex with METTL3/METTL14 (PDB ID: 7NHG). Compound **1** shows a binding mode in which the charged nitrogen of its piperidine group anchors to D395 via a salt bridge. The piperidine moiety pushes the K513 into pocket 4 and opens an aromatic cage consisting of W431, W457, P514, and lipophilic parts of K513, i.e., pocket 6 as for the inhibitor UZH1 (compound **43**; Figure 1). Pocket 6 is also revealed by the MD simulations of the apo METTL3/METTL14, and it shows the possibility for further chemical extensions to an adjacent lipophilic cage from the piperidine of compound **1**. The stability of the lipophilic cage was further confirmed by 300 ns MD simulations on the complex structure of compound **1** and METTL3/METTL14. The root-mean-square deviation (RMSD) values of the residues forming the cage are about 1 Å (Figure S2). The highly lipophilic pocket 6 does not contain any ordered water molecules in the crystal structure. Such a cage mainly has highly unfavorable water molecules in its room-temperature solvated state.^18^ Thus, substituents on the piperidine tail would significantly improve the binding affinity by liberating those unfavorable water molecules.

**Table 1:**
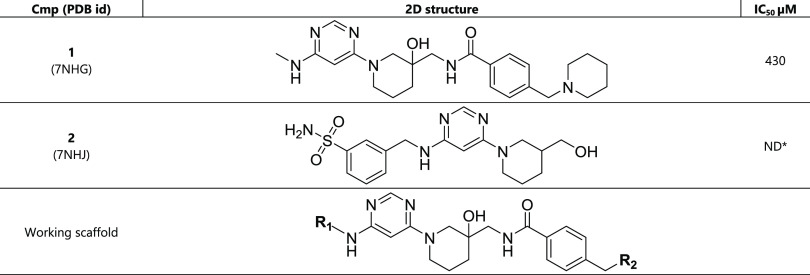
New Scaffolds Identified via Virtual Screening^a^

a In this table and the following ones, the compound concentration at which the METTL3 enzymatic activity was reduced by 50% (IC_50_) was measured by a homogeneous time-resolved fluorescence (HTRF) assay^19^ using the racemic mixtures. ND* = the IC_50_ value could not be measured due to assay interference and/or solubility limit.

### Methyl Groups in Pocket 6 Improve the Binding Affinity

According to the crystal structures and simulation data, we decided to functionalize the piperidine of compound **1**. We first tried lipophilic substituents in position 4 of the piperidine and found that small groups significantly improved the binding affinity; for example, dimethyl attachment in compound **4** shows a 50-fold improvement (Table 2). A few larger lipophilic and polar groups were also considered, but they could not compete with the optimal dimethyl substituent. The improvement in affinity is due to additional van der Waals interactions between the two methyl groups and the hydrophobic side chains in pocket 6 and the release of two to three water molecules.

**Table 2:**
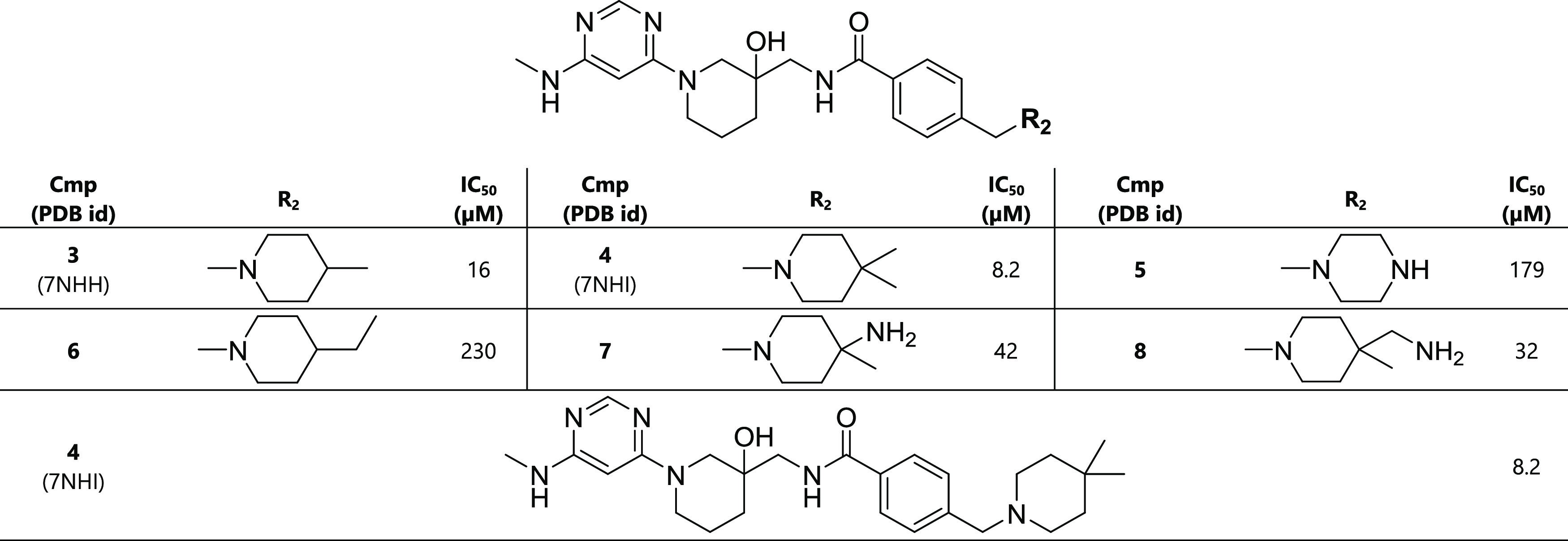
Optimization of Inhibitor Tail (R_2_, Pocket 6)^a^

a The racemic mixture was used for the determination of the IC_50_ value.

### Extending the Inhibitor to Pocket 1 to Form a Cation−π Interaction

**Figure 2:**
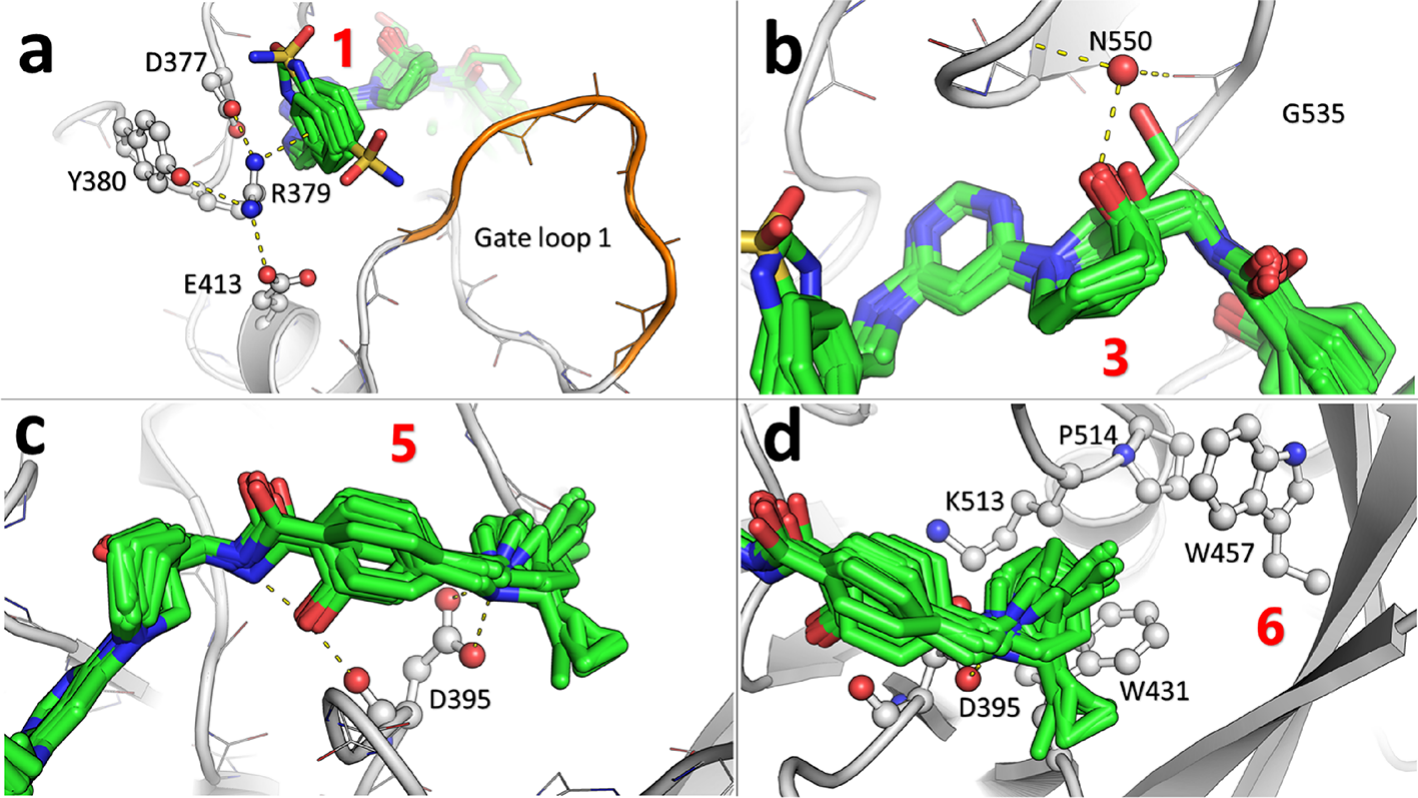
(a–d) Common interactions in pockets 1, 3, 5, and 6 (red labels) as observed by protein crystallography. The crystal structures are 7NHG (**1**), 7NHJ (**2**), 7NHH (**3**), 7NHI (**4**), 7OEL (**9**), 7NHV (**16**), 7NI8 (**29**), 7NI9 (**30**), 7NIA (**31**), 7OEF (**32**), 7NI7 (**51**), 7OEK (**53**), 7OED (**S2**), and 7OEJ (**S5**). The 2D structures of compounds **S2** and **S5** are shown in the Supporting Information.

Compound **2** is another hit identified by virtual screening for which we could solve the crystal structure in complex with METTL3 at a resolution of 2.16 Å (Table 1). The structure (PDB ID: 7NHJ) reveals a solvent-exposed region, i.e., pocket 1, which embraces the benzenesulfonamide group of the compound. This pocket is wrapped by loop 1 and the triple salt bridge D377–R379–E413 (Figure 2a). The crystal structure represents two alternative conformations of the sulfonamide. In one of the conformations, the sulfonamide group interacts with side chains of D377 and N549, and in the other, the sulfonamide group stabilizes loop 1 by interacting with carbonyls of P405, Y406, and T408. Notably, the benzene ring forms a cation−π interaction with R379. MD simulations were used to explore the stability of the interactions observed by the crystal structure. The simulations reproduced the alternative conformations of the sulfonamide group (Figure S3) and confirmed the relatively stable cation−π interaction between the benzene ring and the guanidinium of R379 (Figure 2a). Although the classical force field approximates the cation−π interaction mainly by van der Waals packing, the simulation results suggested preserving this interaction with the side chain of R379. We thus extended compound **4** to pocket 1 by installing a series of aromatic rings on the 4-amino-pyrimidine (Table 3). Large substituents, benzothiophene (**10**) and naphthalene (**15**), improved the affinity by a factor of 10 compared to the reference compound **4**. We decided to prioritize according to ligand efficiency and thus selected for the next round of optimization the benzyl substituent (compound **16**), which has an IC_50_ of 1.7 μM.

**Table 3:**
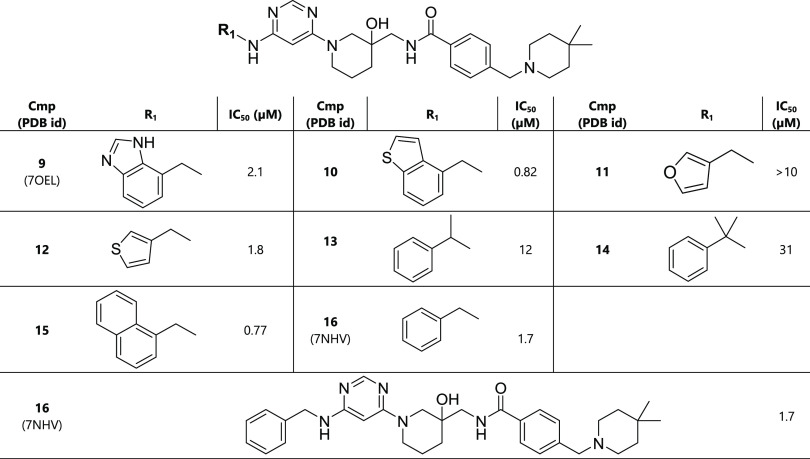
Optimization of the Inhibitor Head-Group (R_1_, Pocket 1)^a^

a The racemic mixture was used for the determination of the IC_50_ value.

### Further Optimization of Interactions in Pocket 6

With the low-μM inhibitor **16** in hand, we decided to further optimize the interactions in the mainly hydrophobic pocket 6 (Table 4). The dimethyl group of the piperidine was first replaced by a cyclopropyl (**17**), 4-ethyl-4-methyl (**18**), and 4-butyl-4-methyl (**19**), but the potency did not improve.

**Table 4:**
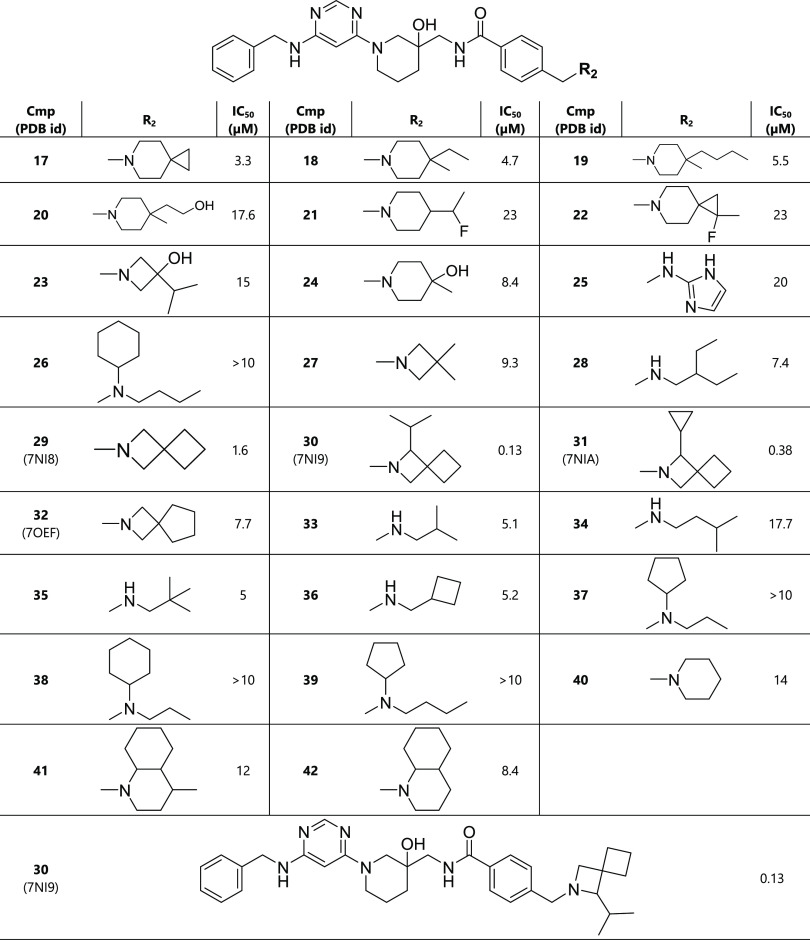
Second Round of Optimization of R_2_ (Pocket 6)^a^

a The racemic mixture was used for the determination of the IC_50_ value.

**Figure 3:**
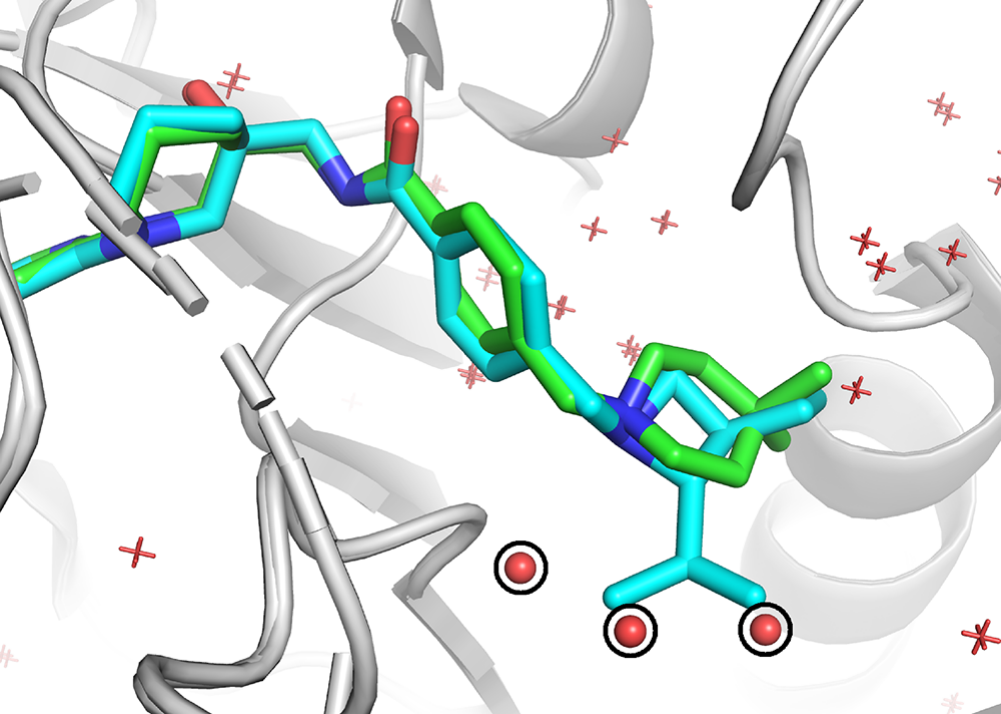
Replacement of crystal water molecules in the half-exposed binding site of pocket 6. The crystal structures of compound **30** (PDB ID: 7NI9) and compound **16** (PDB ID: 7NHV) are superposed and colored cyan and green, respectively. Three crystal water molecules observed in the compound **16**’s crystal structure are shown as the red spheres. These water molecules are replaced by the isopropyl group of compound **30**.

We then considered a set of 26 different substituents (compounds **17**–**42**; Table 4 and Figure 2d). Hydroxyl and fluorine substituents (compounds **20**–**24**) are not tolerated in this region as their binding affinities deteriorate by 5- to 14-fold compared to compound **16**. The replacement of the dimethylpiperidine with 2-amino-imidazole (compound **25**) results also in poorer affinity. These unsuccessful modifications are probably caused by the hydrophobicity of pocket 6. Second, substituents significantly larger than piperidine (compound **26**, IC_50_ > 10 μM) or smaller (compound **27**, IC_50_ = 9.3 μM) worsen the affinity substantially, which is a consequence of poor van der Waals interactions. Third, chain alkane substituents cannot maintain the binding affinity, for example, compound **28**, which is due, at least in part, to the conformational entropy loss upon binding. Fourth, 2-azaspiro[3.3]heptane substituent (compound **29**) shows a slight improvement in binding affinity than piperidine, and an isopropyl attachment at position 1 of the 2-azaspiro[3.3]heptane results in an 13-fold improvement (compound **30**). The comparison of the crystal structures (PDB ID: 7NI9 and 7NHV) shows that the isopropyl displaces three crystal water molecules from the half-open pocket 6 (Figure 3). The remaining compounds (**32**–**42**) showed poorer activity, i.e., a factor of at least 3 worse, than the reference compound **16**.

### Locking the Bound Conformations by Rigidifying the Linker in Pocket 5

**Figure 4:**
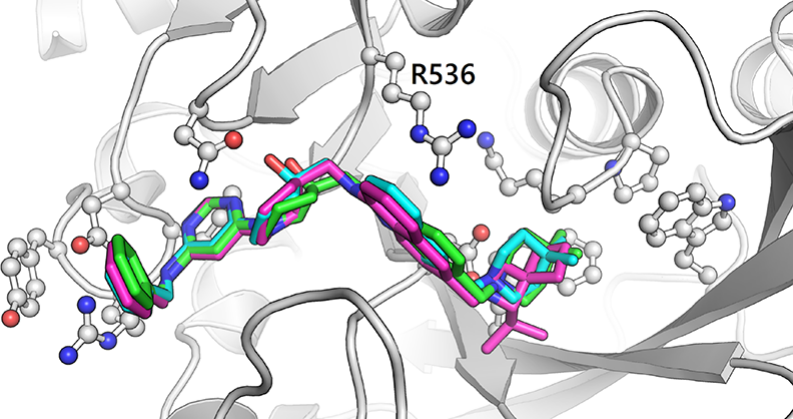
Cation−π interaction formed by R536 and ring systems of compound **46** (cyan, PDB ID: 7NID), **47** (green, PDB ID: 7OEI), and **54** (magenta, PDB ID: 7OQL) in pocket 5.

In parallel to the piperidine modification, we optimized the benzamide linker bound in pocket 5 by starting from the reference compound **16** (Table 3). The structural superposition shows that the benzamide can rotate around the linker’s rotation axis and thus offers flexibility in pocket 5 (Figure 2c,d). Restriction of the rotation may contribute to the binding affinity due to a reduced loss of ligand conformational entropy upon the binding. We first modified the para-position of the benzamide and aimed to form an intramolecular hydrogen bond. Phenol (compound **43**) and thioanisole (compound **44**) were used to replace the benzene ring (Table 5). The thioanisole replacement keeps the binding pose and maintains the binding affinity. By comparison, the phenol replacement increases the binding affinity by 4-fold compared to compound **16**. The crystal structures show that the added hydroxyl group not only forms an intramolecular hydrogen bond with −NH of the amide but also interacts with the backbone of D395 as the hydrogen-bond donor (Figure 2c). We then tried the ring fusion strategy to restrain the ligand conformation in its bound-like state. The aromatic 1-naphthylamine and benzimidazole (compounds **46** and **47**, respectively) and a spiro scaffold (compound **48**) (Table 5) were used for this purpose. The substitutions as in **47** and **48** do not improve the binding affinity, while **46** improves the binding by 5-fold. According to the crystal structure of inhibitor **46** (PDB ID: 7NID), the 1-naphthylamine successfully locks the conformation (Figure 4) and closely contacts R536 with a plausible cation−π interaction.

**Table 5:**
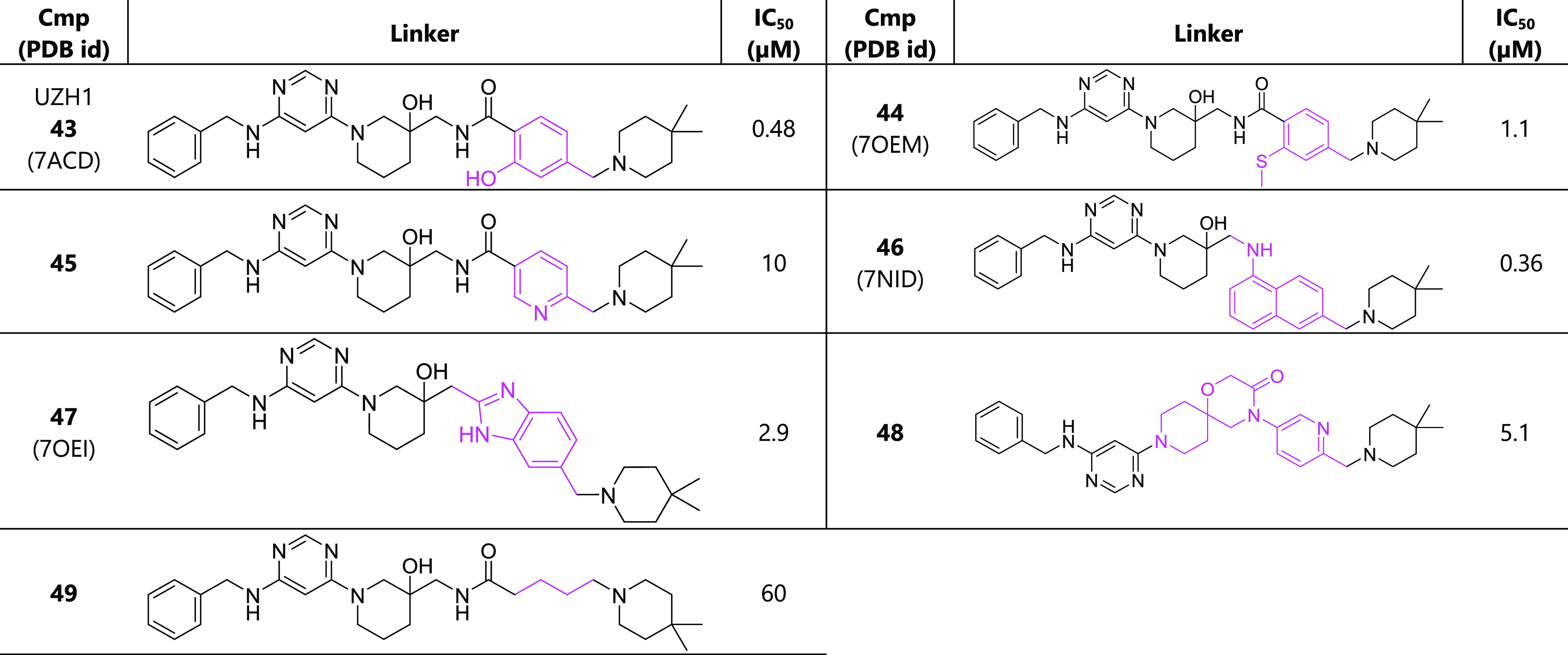
Optimization of the Linker (Magenta) Connecting to the Dimethylpiperidine in Pocket 5^a^

a The racemic mixture was used for the determination of the IC_50_ value.

### Combination of Potent Structural Features

We first replaced in the reference compound **16** the dimethylpiperidine with the optimal substituent for pocket 6 (i.e., the 1-isopropyl-2-azaspiro[3.3]heptane of compound **30**) and phenolic linker (of compound **43**). The resulting compound **50** shows poorer binding (IC_50_ = 0.27 μM) than inhibitor **30** (IC_50_ = 0.13 μM). To understand the failure, we compared two crystal structures whose bound inhibitors contain the common functional group isopropyl-azaspiro-heptane. One compound has the phenyl linker (**30**, PDB ID: 7NI9), and the other contains the phenol instead (**S7**, PDB ID: 7OQP). We find that the compound with the phenolic hydroxyl group reorients the linker compared to that with phenyl and, in turn, introduces inevitable clashes between the linker and its isopropyl-azaspiro-heptane group. We also combined the phenol linker with other substituents in P1 or P6 (compounds **50**–**53** in Table 6 and **S1**–**S8** in Table S1), but none of them did improve the binding significantly.

**Table 6:**
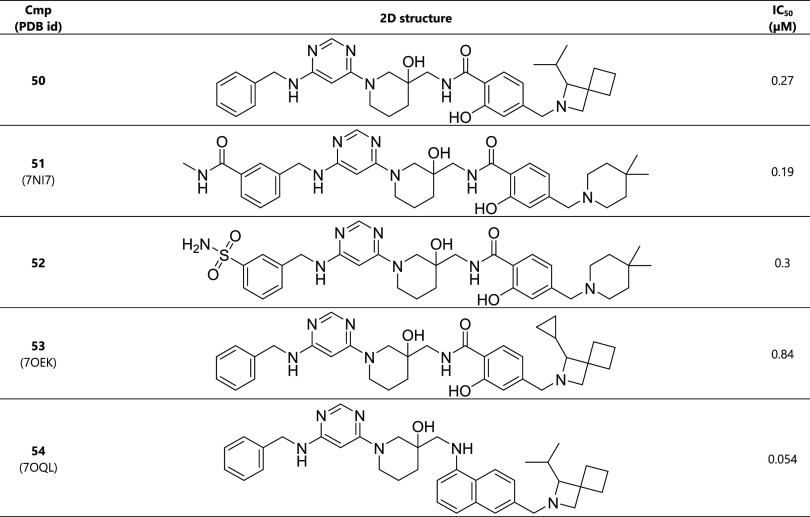
Combination of Potent Substituents^a^

a The racemic mixture was used for the determination of the IC_50_ value.

When combining the isopropyl-azaspiro-heptane tail and the 1-naphthylamine linker, we achieved a significant improvement in affinity with compound **54** (IC_50_ = 54 nM). Thus, starting from the hit compound **1** (IC_50_ = 430 μM), we have improved the binding affinity by approximately 8000-fold as measured in the enzymatic assay. The crystal structure of the complex of METTL3 with inhibitor **54** (PDB ID: 7OQL) shows that the naphthalene aligns well with the benzamide (Figure 4), successfully locking the binding conformation without perturbing its environmental interactions much. Furthermore, the isopropyl-azaspiro-heptane favorably interacts with the aromatic cage, i.e., pocket 6.

To shed light on the 8000-fold improvement in affinity for METTL3 of lead compound **54** vs hit **1**, it is useful to analyze the matched pairs. The replacement of the methyl at R_1_ by phenyl results in a factor of 4.8 improvement (matched pair **4**:**16**), while the change of the benzamide linker by 1-naphthylamine improves the affinity by a factor of 4.7 (matched pair **16**:**46**). The largest improvement in potency is due to the optimization of the R_2_ group in the hydrophobic pocket 6, which corresponds to a factor of 52.4 times 6.7 (matched pairs **1**:**4** and **46**:**54**, respectively). Considering these four matched pairs as independent one obtains an 7920-fold (4.8 × 4.7 × 52.4 × 6.7) improvement in affinity, which is very close to the one of the matched pair hit **1**:lead **54** (7963-fold). Thus, the analysis of the matched pairs provides evidence that the three modifications (R_1_ in pocket 1, linker in pocket 5, and R_2_ in pocket 6, respectively) are additive. This observation is intuitively consistent with the linear shape of this series of compounds and their extended conformation in the bound state.

We then decided to evaluate the cellular potency of inhibitor **54**. The antiproliferative activity on MOLM-13, which is an acute myeloid leukemia cell line, was measured using a previously published protocol with a 3-day incubation time.^10^ Dose–response measurements were carried out to determine the concentration of the inhibitor, which results in 50% inhibition of cellular growth (GI_50_). Inhibitor **54** has GI_50_ = 6 μM for MOLM-13 (Figure S7). The cellular GI_50_ value is higher than the biochemical IC_50_ value by a factor of about 100, which is due, at least in part, to the high concentration of the co-substrate SAM in the cell (60 to 160 μM in the rat liver).^20^

### Rationalize the Binding Difference between *R* and *S* Configurations of the Piperidine Scaffold

The chirality of the piperidine-3-ol piece is essential for the binding of all the compounds reported here (Figure 2b). The *R* configuration shows a stronger binding affinity compared to its *S* counterpart for the seven compounds (**16**, **29**, **31**, **43**, **46**, **S1**, and **S2**) for which the two enantiopure compounds were obtained (Table S2). The *R* configuration prevails over the *S* one by a factor of 3 to 100. As expected, the inhibition of the enzymatic activity of the racemic mixture is slightly poorer than that of the *R* configuration.

**Figure 5:**
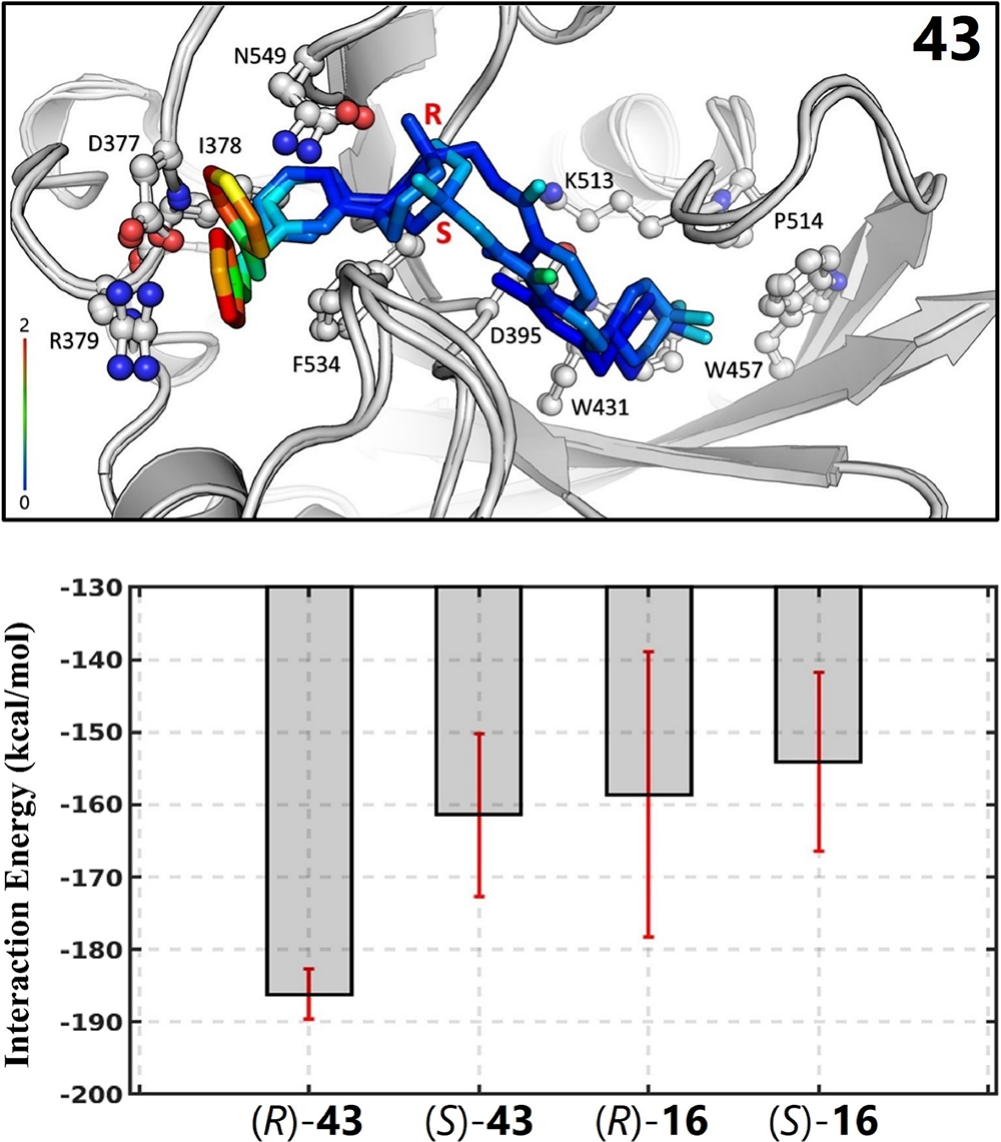
Understanding binding mechanisms of *R*/*S* configurations of UZH1 (**43**). Top: Binding poses of *R* (PDB ID: 7ACD) and *S* (modeled) configurations of UZH1. Ligands are shown in the sticks and colored according to their RMSF values. Interacting residue side chains are represented in balls and sticks. Bottom: Interaction energies of UZH1 and compound **16** with the METTL3/METTL14 complex. For the calculations, interactions between ligands and their surroundings were considered, including proteins, water molecules, and salt ions. Each interaction energy value was averaged by five replicas, and the uncertainty was estimated by their standard error of the mean values.

Compound **43** (UZH1a) shows an IC_50_ of 4.6 μM for m^6^A/A reduction in MOLM-13 cells.^21^ Its *R* configuration is approximately 100-fold more active than its *S* counterpart. Thus, understanding the critical difference would help further optimization. We were not successful in solving the complex structure of METTL3 with the less active *S* configuration. To determine its binding mechanism to METTL3, we modeled its binding mode using the crystal structure of compound (*S*)-**S2** (PDB ID: 7OEE, 2D structure in the Supporting Information) as the template for an *S* configuration. The binding poses of *R* and *S* configurations show different interactions with the catalytic site (Figure 5). First, the *S* configuration’s benzamide linker moves out of pocket 4 relative to its *R* counterpart because of chiral changes of the piperidine-3-ol piece, making a compound more exposed to loop 1. Second, the piperidine of *S* configuration in pocket 2 has seemingly weaker hydrogen-bond interactions with the backbone NH of I378 than that of *R* configuration. The HB donor–acceptor distance reflects the attenuated hydrogen-bond interactions, i.e., the heavy atoms of backbone N and N1 of the piperidine. For example, compound **43** has a distance of 3.0 and 3.5 Å for its *R* and *S* configurations, respectively. In addition, the benzyl group of *S* configuration in pocket 1 loses the well-defined cation−π interaction with R379 compared to that of *R* configuration.

We then conducted comparative MD simulations for the *R* and *S* configurations of compound **43** in their bound form. The two configurations mostly show stable binding to the catalytic site, which is revealed by comparing their mapped RMSD values (Figure 5). The benzene ring in pocket 1 frequently rotates for the *R* configuration and dynamically forms a cation−π interaction with R379. By contrast, the benzene ring of the *S* configuration loses the cation−π interaction and initiates an edge-to-face contact with the R379–D377 plane (Figure 5). To understand the origin of the binding difference between the two configurations, we calculated the interaction energies between the bound inhibitors and their interactive environment, including protein residues, water molecules, and salt ions. The calculations clearly show that the *R* configuration interacts with its surroundings more favorably than the *S* configuration (the bottom in Figure 5). As the reference, we carried out the same calculation for compound **16**, which only has approximately a 6-fold difference between its *R* and *S* configurations. As shown in Figure 5, its two configurations do not show a significant difference in their interactions with the rest of the systems.

The interaction energy analysis can explain only part of the substantial difference between *R* and *S* configurations of an inhibitor (Figure 5). We further analyzed the conformational preference of the inhibitors. To this end, we chose several representative compounds in Table S2, in which their bound states of *R* and *S* configurations can be reliably built based on the crystal structures. We calculated ligand strain energies (see the Materials and Methods section) for these inhibitors and compared the difference between their *R* and *S* counterparts via the Freeform module implemented in OpenEye.^22^ The simulations show that the strain energy of an *R* configuration always prevails relative to its *S* counterpart irrespective of comparing their local or global strain energies (Table S4). This result suggests that, upon binding, the *R* configuration undergoes less energy penalty than its *S* enantiomer.

### Selectivity of the METTL3 Inhibitor **54**

To investigate the selectivity of the most potent inhibitor (compound **54**, IC_50_ = 54 nM) toward other RNA methyltransferases, we tested it using the thermal shift assay against METTL1 and METTL16. METTL1 mediates the formation of N7-methylguanine in a subset of RNA species, such as tRNAs, mRNAs, and microRNAs, whereas METTL16 is able to N6-methylate a subset of mRNAs and U6 small nuclear RNAs.^23,24^ The co-product of methyl transfer catalysis, *S*-adenosyl-l-homocysteine (SAH), was used as a positive control in the thermal shift assay. SAH showed Δ*T*_m_ of 4.05, 8.75, and 2.5 °C at 2000 μM for METTL3/METTL14, METTL1, and METTL16, respectively. Compound **54** at 200 μM was able to shift the melting curve of METTL3/METTL14 by 1.8 °C compared to the DMSO control (Figure S4). No shift was observed for METTL1 and METTL16 with compound **54** up to 200 μM (Figures S5 and S6).

## Conclusions

Starting from a high micromolar METTL3/METTL14 inhibitor identified by high-throughput docking (hit **1**), we have carried out a medicinal chemistry optimization campaign supported by protein X-ray crystallography and MD simulations. The optimization of the hit was carried out on three of its groups independently (extension into pocket 1, linker in pocket 5, and hydrophobic moiety in pocket 6, respectively). An analysis of matched pairs revealed that the three independent improvements were additive. The largest contribution to the overall 8000-fold enhancement of the inhibitory activity of lead **54** vs hit compound **1** originated from the optimization of the group in the hydrophobic pocket 6. The structures of the complexes of METTL3/METTL14 with small-molecule inhibitors provided a high-resolution description of the most common interaction motifs with the SAM pocket. The MD simulations revealed flexibility of the loops at the entrance of the SAM pocket and provided an explanation for the difference in the affinity of the *R* and *S* configurations of the piperidine scaffold in pocket 3. Compound **54** shows an IC_50_ of 54 nM for METTL3/METTL14 in the HTRF assay, selectivity against the off-targets METTL1 and METTL16, and a GI_50_ of 6 μM for the MOLM-13 AML cell line.

## Materials and Methods

### Protein Expression and Purification

Recombinant METTL3/METTL14 complex constructs for crystallization and for the use in the enzymatic activity assay were expressed using the baculovirus/Sf9 insect cell expression system, as described previously.^17^

### Crystallization

The SAH (*S*-adenosyl-l-homocysteine)-bound holo protein crystals of METTL3_354–580_–METTL14_106–396_ were obtained, as previously described.^25^ The compounds to be tested were dissolved in DMSO at concentrations of 50–200 mM depending on their solubility. Protein–ligand complex structures were solved by soaking compounds into holo protein crystals replacing the bound SAH in the METTL3 catalytic pocket. The experiment was carried out by evaporating the solvent (DMSO) overnight from the drop. First, 1 μL of the compound dissolved in DMSO was left overnight to evaporate the solvent. The next day, 1 μL of mother liquor containing 30% PEG-3350 and 200 mM Mg acetate was added on top of the dried compound stamp. One holo crystal was then transferred into the mother liquor over the target compound stamp. After 16 h of incubation at 22 °C, the crystals were harvested and flash-frozen in liquid nitrogen.

### Data Collection and Structure Solution

Diffraction data were collected at the PXIII beamline at the Swiss Light Source (SLS) of the Paul Scherrer Institute (PSI, Villigen, Switzerland) and processed using XDS.^26^ The crystal structures were solved by molecular replacement techniques using the 5L6D structure as the search model with the Phaser program from the Phenix package.^27^ In the crystals not subjected to soaking, clear electron density for product cofactor *S*-adenosyl-homocysteine (SAH) is visible. Therefore, in this soaking experiment setup, test compounds competed with SAH for the *S*-adenosyl methionine (SAM) binding site. In the crystal structures of adenosine analogues that were able to replace SAH in the binding site, the electron density due to the homocysteine part of SAH was no longer visible. All of the crystallographic models were constructed through iterative cycles of manual model building with COOT and refinement with phenix.refine.^27,28^

### Enzymatic Assay (HTRF)

Compound potencies were evaluated by using a previously reported METTL3 inhibition assay. Briefly, the level of m^6^A in the oligoribonucleotide substrate after the reaction catalyzed by METTL3/METTL14 was quantified by measuring specific binding of modified oligoribonucleotide to the m^6^A reader YTHDC1_345–509_ by HTRF. Tested compounds that inhibit METTL3 decrease the m^6^A level and thus reduce the HTRF signal. The biochemical assay was performed, as described previously.^19^ The IC_50_ values derived from fitting a dose–response curve to the data using nonlinear regression. The IC_50_ values are given as an average of at least two independent measurements for each compound in the text.

### Thermal Shift Assay

The protein sample was buffered in 25 mM HEPES (pH 7.4) and 150 mM NaCl and assayed in a 96-well plate at a final concentration of 2 μM in 20 μL volume. The SYPRO Orange dye was added as a fluorescence probe at a dilution of 1:1000. The compound **54** concentrations tested were 10, 50, 100, and 200 μM, while the SAH concentrations tested were 100, 500, 1000, and 2000 μM. The temperature was raised with a step of 0.5 °C starting from 20 to 80 °C, and fluorescence readings were taken at each interval. The reported values (Δ*T*_m_) are calculated as the difference between the transition midpoints of an individual sample and the average of the reference wells (containing the protein and the DMSO only) in the same plate. The DMSO concentration was kept at 1% (v/v).

### Cell Culturing

MOLM-13 cells were cultured in RPMI 1640 medium containing 10% Gibco FBS and 1% penicillin/streptomycin (complete medium) in 5% CO_2_ at 37 °C in a humidified incubator, with maintained cell densities at 0.6–2 × 10^6^ cells/mL.

### Cytotoxicity (GI_50_)

Cells were seeded in white clear-bottom 96-well plates at a density of 2 × 10^4^ cells/well in 50 μL of the complete RPMI medium and treated with 50 μL increasing concentrations of compound **54** dissolved in DMSO (final concentration of compounds 1.25–160 μM) or DMSO only (0.5% (v/v)) as a negative control and incubated for 72 h at 37 °C with 5% CO2. Cell viability was determined using a CellTiter-Glo luminescent cell viability assay (Promega G7570) based on the detection of ATP according to the manufacturer’s instructions. The reagent (100 μL) was added to each well and incubated for 10 min at room temperature on an orbital shaker. The luminescence was recorded using a Tecan Infinite 3046 M1000 microplate reader from the top. The background luminescence value was obtained from wells containing the CellTiter-Glo reagent and medium without cells. Cell viability curves were plotted in GraphPad Prism 9 and fitted with nonlinear regression, from which GI_50_ values were determined.

### Chemical Synthesis

#### Synthesis Route of Compound **54**

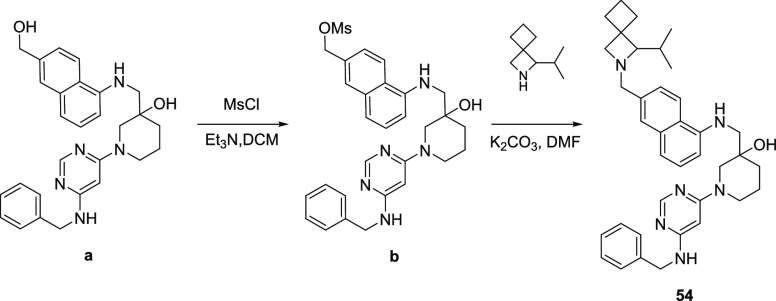



#### Synthesis of Compound **b**

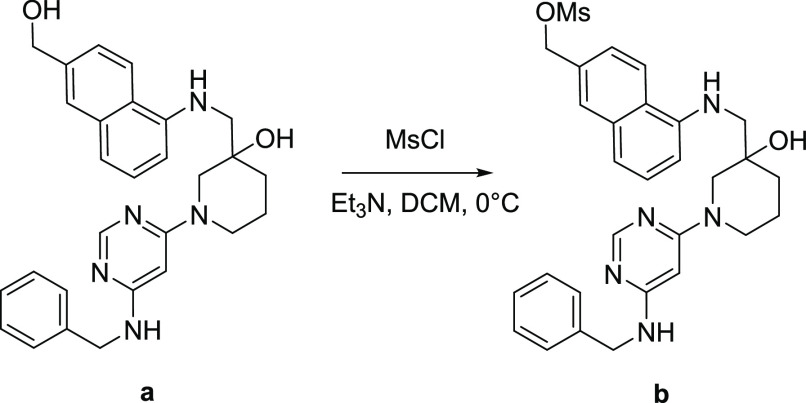



To a solution of 1-(6-(benzylamino)pyrimidin-4-yl)-3-(((6-(hydroxymethyl)naphthalen-1-yl)amino)methyl)piperidin-3-ol (300 mg, 0.64 mmol) in dichloromethane (5 mL) were added MsCl (88 mg, 0.76 mmol) and Et_3_N (97 mg, 0.95 mmol) at 0 °C, and the mixture was stirred at 0 °C for 2 h. The TLC (5% MeOH in dichloromethane, *R_f_* = 0.5) showed a new spot and no start material. The mixture was concentrated to give (5-(((1-(6-(benzylamino)pyrimidin-4-yl)-3-hydroxypiperidin-3-yl)methyl)amino)naphthalen-2-yl)methyl methanesulfonate as a white solid (340 mg, crude).

#### Synthesis of Compound **54**

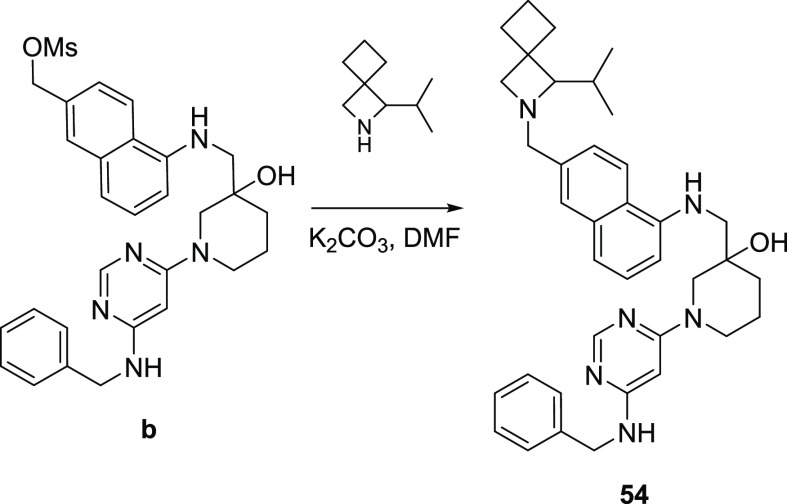



To a solution of (5-(((1-(6-(benzylamino)pyrimidin-4-yl)-3-hydroxypiperidin-3-yl)methyl)amino)naphthalen-2-yl)methyl methanesulfonate (170 mg, 0.31 mmol) in DMF (3 mL) were added 1-isopropyl-2-azaspiro[3.3]heptane hydrochloride (47 mg, 0.34 mmol) and K_2_CO_3_ (55 mg, 0.40 mmol), and then the mixture was stirred at 25 °C for 16 h under N_2_. LCMS showed that the reaction was completed. The mixture was concentrated and purified by HPLC to give the desired product 1-(6-(benzylamino)pyrimidin-4-yl)-3-(((6-((1-isopropyl-2-azaspiro[3.3]heptan-2-yl)methyl)naphthalen-1-yl)amino)methyl)piperidin-3-ol as a white solid (15 mg, yield: 8%).

### MD Simulations and Analysis

#### Model Building

Eleven molecular systems for MD simulations were constructed based on previously published structures and newly released ones in this study. These systems include one apo system (METTL3/METTL14 complex), six holo systems (METTL3/METTL14 complex bound with different inhibitors), and four ligands in the unbound state. Their general information is also summarized in Table S3. We mainly take the apo system as an example to describe the model-building procedure, and the difference of model buildings for other systems will be highlighted if necessary.

The original coordinates for the apo system, including proteins and crystal water molecules, were extracted from the structure 5K7M.^11^ The original sequence length was kept for the following treatment, i.e., METTL3 ranging from residues 369 to 570 and METTL14 from residues 116 to 399. The protonation states of residues were first determined by PropKa-3.0 and manually checked afterward.^29^ Specifically, His512 of METTL3 was flipped and protonated to form a salt bridge with Asp395 according to the suggestion by the structure 5IL0.^13^ The hydrogen atoms were then added by the CHARMM program (version 42b2).^15^ For those residues with alternative conformations, the first suggested coordinates in the PDB file were used. Missing atoms for certain residues were added according to IC tables of the CHARMM topology file. The METTL3/METTL14 complex system was solvated in a rhombic dodecahedron (RHDO) TIP3P water box (lattice length: 107 Å) to ensure at least 10 Å buffer space between the protein atoms and the boundary of the water box. To neutralize the system and mimic the physiological conditions, Na^+^ and Cl^–^ ions at a 0.15 M concentration were added to the solvated systems. Finally, the entire system includes 83,104 atoms in total.

Six holo systems (bound with inhibitors) were trimmed similarly compared to the apo system with a bit varied number of water molecules and salt atoms. The main coordinates of each system were extracted from its corresponding X-ray structure (Table S3), including METTL3/METTL14, crystal water molecules, and inhibitors. The missing loops, i.e., gate loop 1, were built by SWISS-MODEL,^30^ and other missing structural components, if any, were created based on 5K7M. The sequence length of the proteins was kept the same as the apo system. Because we could not solve the structure of METTL3/METTL14 in complex with inhibitor **43***S*, we built it based on its analogue structure **S2** (7OEE), whose complex structure with its *S* configuration had been successfully determined. The models of **16***R* and **16***S* were built based on those of **43***R* and **43***S*, respectively, by only removing the hydroxyl from the phenyl.

#### MD

Each simulation system, taking the apo system as an example, was initially minimized for 10,000 steps with a series of constraints and restraints on the solute molecules to release its bad contacts and poor geometries. The minimized structure was heated to 300 K and equilibrated in an NVT condition (constant volume and temperature). The equilibrated system was continually heated to 600 K to enhance the sampling of water molecules and ionic atoms. The system was then cooled down to 300 K. All heavy atoms of the solute molecules were restrained with harmonic potentials during the simulations at the NVT condition. Finally, the structure was further equilibrated in an NPT condition (constant pressure and temperature) with weak restraints on the backbones of the proteins. All the equilibration phases lasted for 1 ns using the CHARMM program (version 42b2).^15^ A 500 ns of the production run was carried out in NPT conditions using the NAMD program (version 2.13) without any restraints on the systems.^16^ The pressure was controlled by the Nosé–Hoover Langevin piston method with a 200 ps piston period and a 100 ps piston decay time.^31,32^ The temperature was maintained at 300 K using the Langevin thermostat with a 5 ps friction coefficient. The integration time step was set to 2 fs by constraining all the bonds involving hydrogen atoms by the SHAKE algorithm. Van der Waals energies were calculated using a switching function with a switching distance from 10 to 12 Å,^33^ and electrostatic interactions were evaluated using the particle mesh Ewald summation method.^34^ Lennard-Jones long-range correction was enabled.^35^ Other holo and ligand systems were conducted with a similar protocol, and necessary information is listed in Figure S1. To ensure sufficient sampling, all the systems were sampled in independent runs, namely, replicas, with random initial velocities.

The CHARMM36m force field was used for proteins,^36^ and organic molecules were parameterized by CGenFF.^37^ MD snapshots were saved every 20 ps along the MD trajectories for further analysis. Geometric measurements, for example, RMSD and atomic distance analyses, were performed with CHARMM routines. All statistical figures were plotted by MATLAB (version 2018a),^38^ and structural figures were generated with the PyMOL graphic software (version 2.3).^39^ Maestro (version 11.5) was used for analyzing protein–ligand interactions and trim models.^40^

#### Calculations of Conformer (Ligand) Strain Energies

The ligand strain energies of the compounds in Table S4 were calculated by the Freeform module implemented in OpenEye.^41^ During the step of conformational search, at most 20,000 configurations were allowed to generate via the conformation generator OMEGA in the Sheffield solvation model.^42^ After minimization of all conformers, the unique conformers were kept by removing redundant ones. The partition function for the minimized conformer ensemble was calculated by considering conformer entropies.^43^ The Helmholtz conformer free energy was then calculated for each conformer. To calculate the ligand strain energies, we modeled binding conformers for both *R* and *S* configurations of each compound based on the corresponding crystal structures and then submitted them to Freeform for restrained and unrestrained minimizations. Based on the minimization results and the obtained partition function, the local and global strain energies for each configuration (*R* or *S*) were calculated. All the results are listed in Table S4.

## Supporting Information Available

Supplementary figures and tables, chemical structures of compounds **S1**–**S8**, and characterization of inhibitor **54**, which includes the ^1^H and ^13^C NMR spectra and the HPLC purity trace (PDF)

## Accession Codes

7NHG (**1**), 7NHJ (**2**), 7NHH (**3**), 7NHI (**4**), 7OEL (**9**), 7NHV (**16**), 7NI8 ((*R*)-**29**), 7OEG ((*S*)-**29**), 7NI9 (**30**), 7NIA ((*R*)-**31**), 7OEH ((*S*)-**31**), 7OEF (**32**), 7OEM (**44**), 7NID (**46**), 7OEI (**47**), 7NI7 (**51**), 7OEK (**53**), 7OQL (**54**), 7OED ((*R*)-**S2**), 7OEE ((*S*)-**S2**), 7OEJ (**S5**), 7OQO (**S6**), 7OQP (**S7**).

## Notes

All compounds showed ≥95% purity according to HPLC analysis.
