# Supplementary material for: Structure-Based Design of Inhibitors of the m6A-RNA Writer Enzyme METTL3
Source: ACS Bio Med Chem Au. 2023 Jun 14;3(4):359–70. doi: 10.1021/acsbiomedchemau.3c00023 (PMC10436262; doi:10.1021/acsbiomedchemau.3c00023)
Supplement: Supplementary file 1 — bg3c00023_si_001.pdf [file bg3c00023_si_001.pdf]

Structure-based design of inhibitors of the m^{6}A-RNA writer enzyme METTL3

Rajiv Kumar Bedi, Danzhi Huang, Yaozong Li, Amedeo Caflisch*

Department of Biochemistry, University of Zurich, Winterthurerstrasse 190, Zurich CH-8057, Switzerland

*Email: caflisch@bioc.uzh.ch. Phone: +41 44 635 55 21

SUPPORTING INFORMATION

Table of contents

Figures 2

Tables 9

NMR and HPLC traces 12

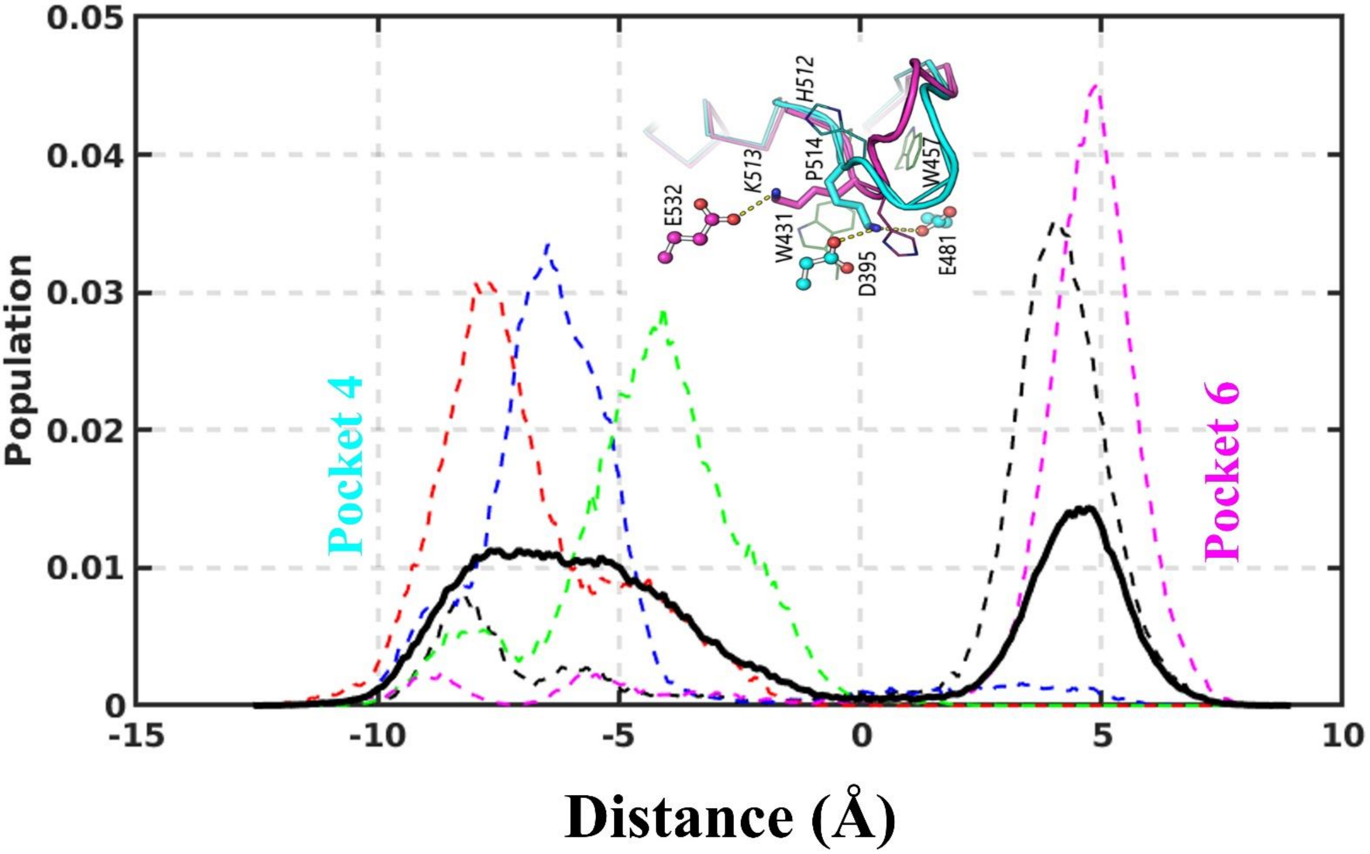

Figure S1: Distance distribution for monitoring the occupancy of pockets 4 and 6 by the side chain of K513 in the simulations of apo METTL3/METTL14. The distance is defined as $d=d_1–d_2$, here, d_1: K513 (NZ) to E481 (CD); d_2: K513 (NZ) to E532 (CD). The probability distribution is binned with a width of 0.2 Å. Distribution of individual trajectories are shown by dashed lines of different colors while the total sampling is shown by the solid black line.

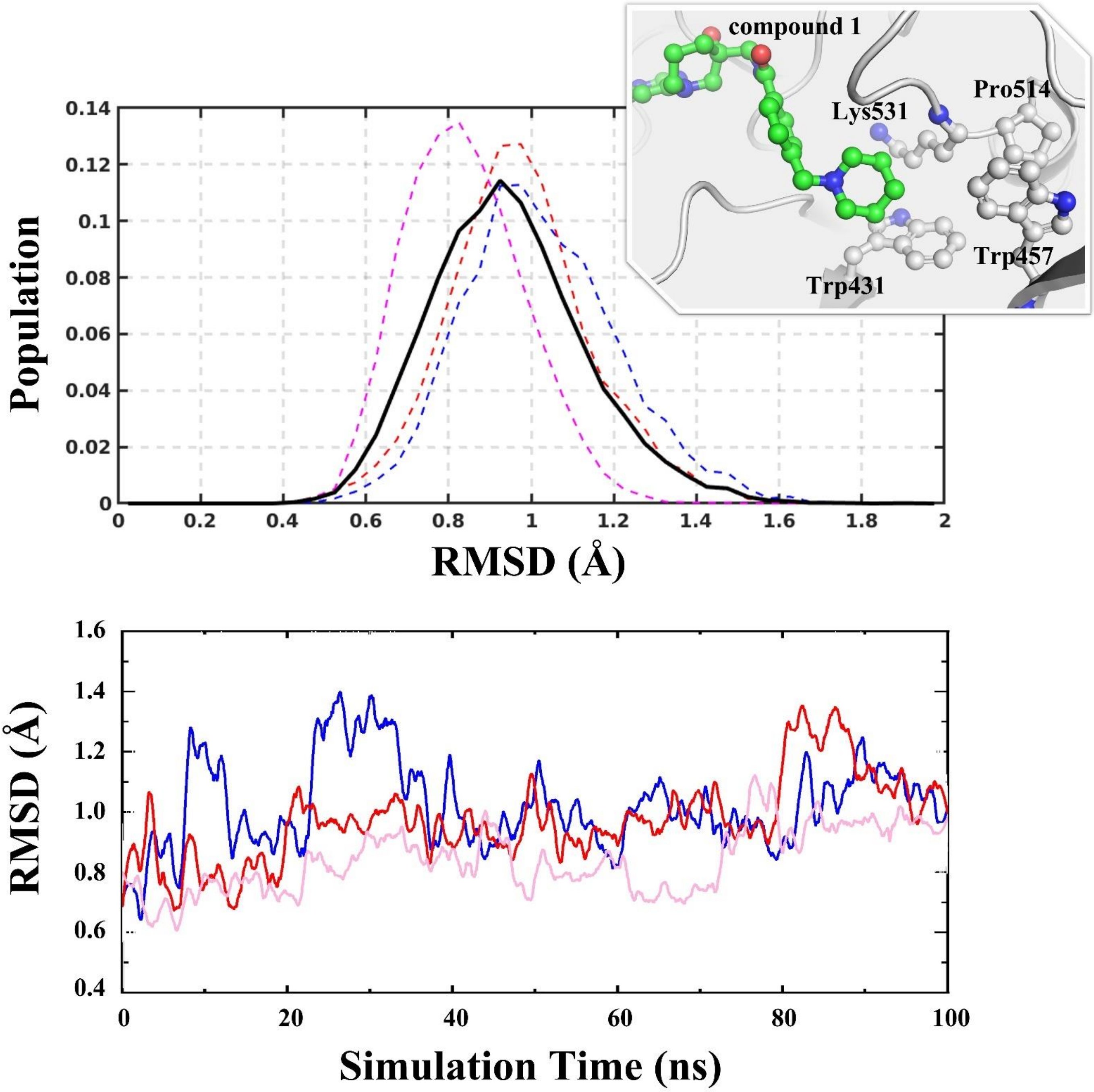

Figure S2: Distribution and time series of RMSD values of the residues constituting the aromatic cage for the simulations with compound **1**. The residues W431, W457, P514, and K531 (gray balls and sticks) were used for calculating the root mean square deviation (RMSD). The minimized crystal structure was used as the reference structure, and snapshots from 300 ns MD simulations (100 ns for each replica) were superposed to the reference structure. The histogram was binned with a width of 0.1 Å and normalized to obtain the population density values. The distribution for the three trajectories is plotted in the solid black line, and the one for each replica is plotted in dashed lines.

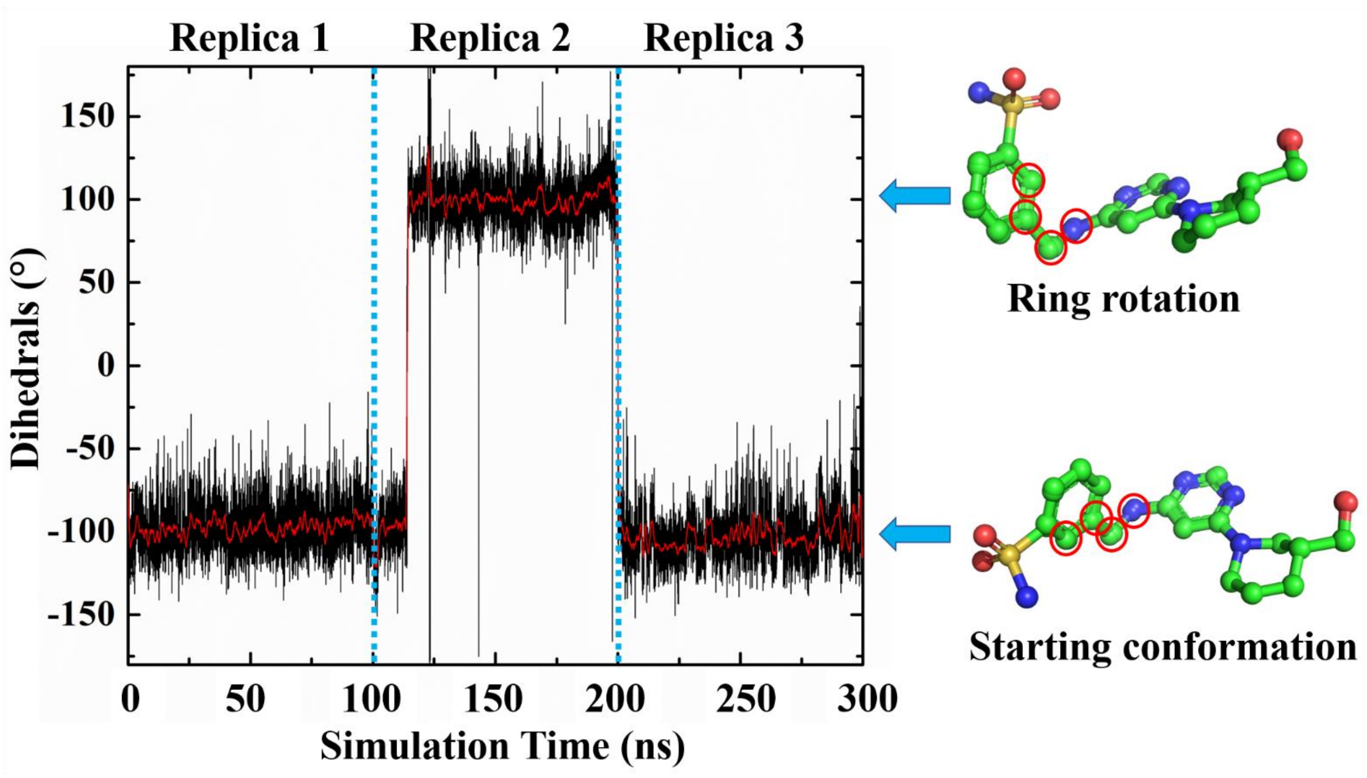

Figure S3: Flipping of the benzene sulfonamide of compound **2**. Red circles label the measured dihedral angle. The time series is smoothed by the adjacent-averaging method (red lines). Three independent MD simulations of 100 ns each were carried out (separated by vertical dashed lines in cyan). The simulations started with a value of the dihedral angle of approximately -100°. The benzene sulfonamide rotated in replica 2 to about +100°.

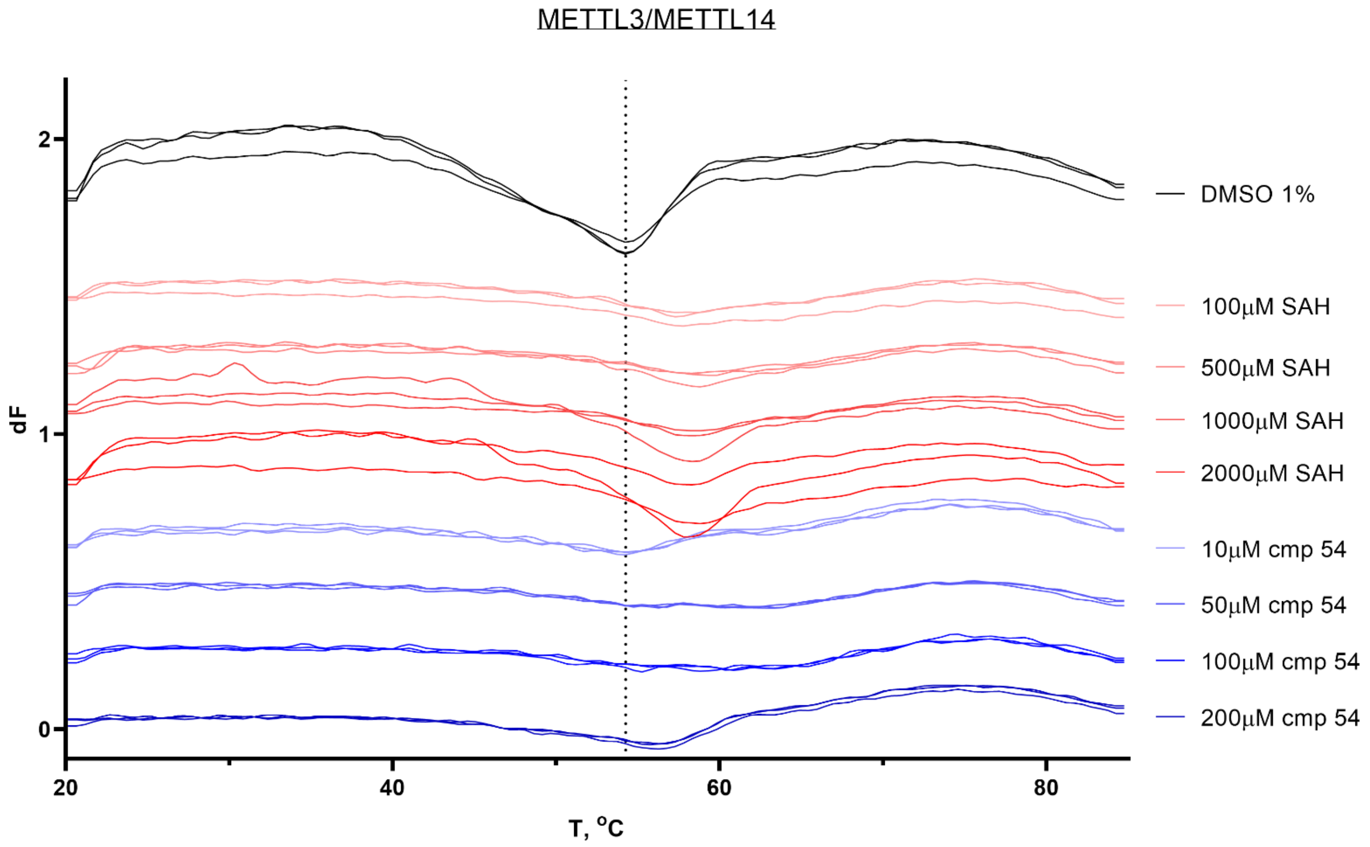

Figure S4: METTL3/METTL14 thermal shift assay. Shown are the first derivative of the melting curves of METTL3/METTL14 in the presence of inhibitor **54** or SAH at different concentrations.

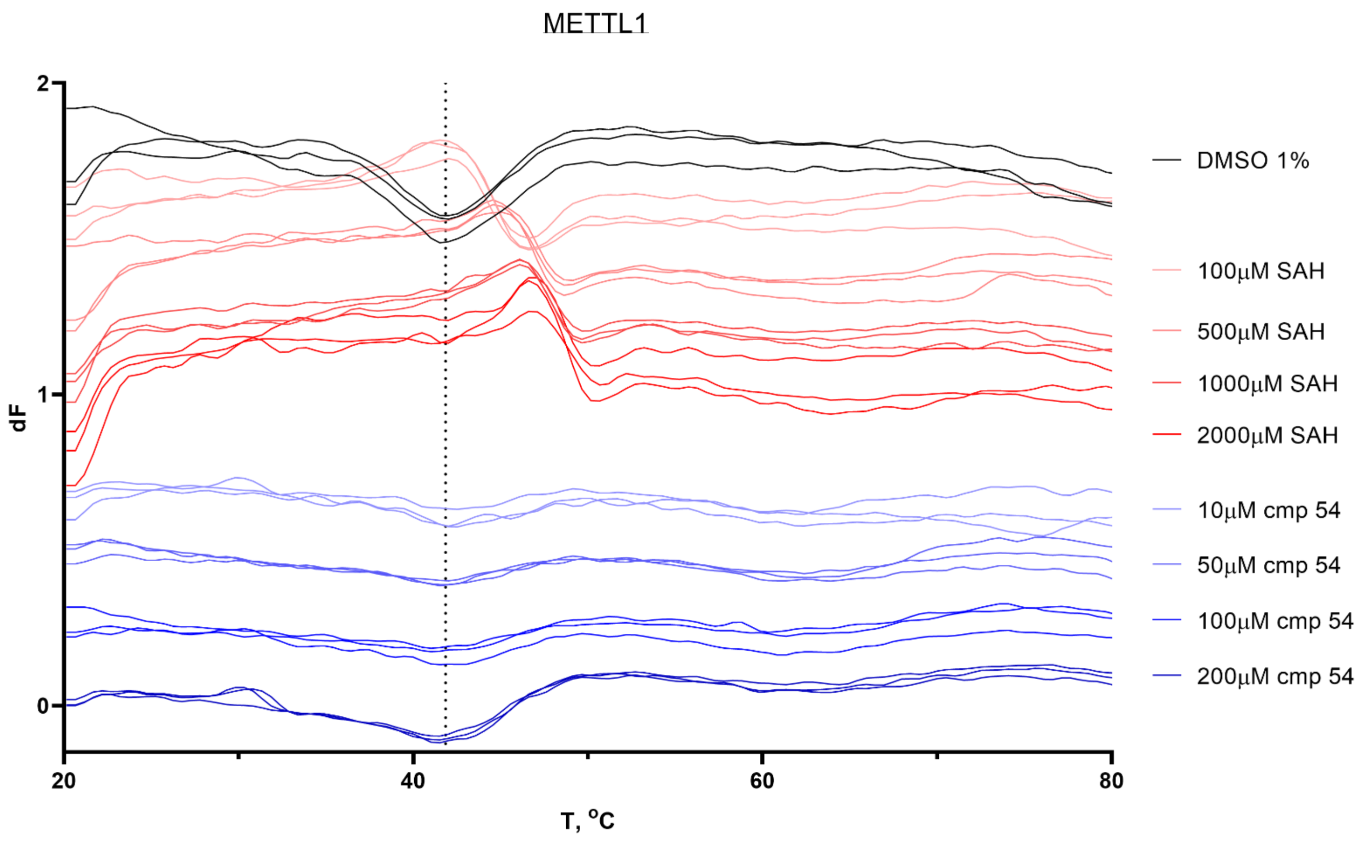

Figure S5: METTL1 thermal shift assay. Shown are the first derivative of the melting curves of METTL1 in the presence of inhibitor **54** or SAH at different concentrations.

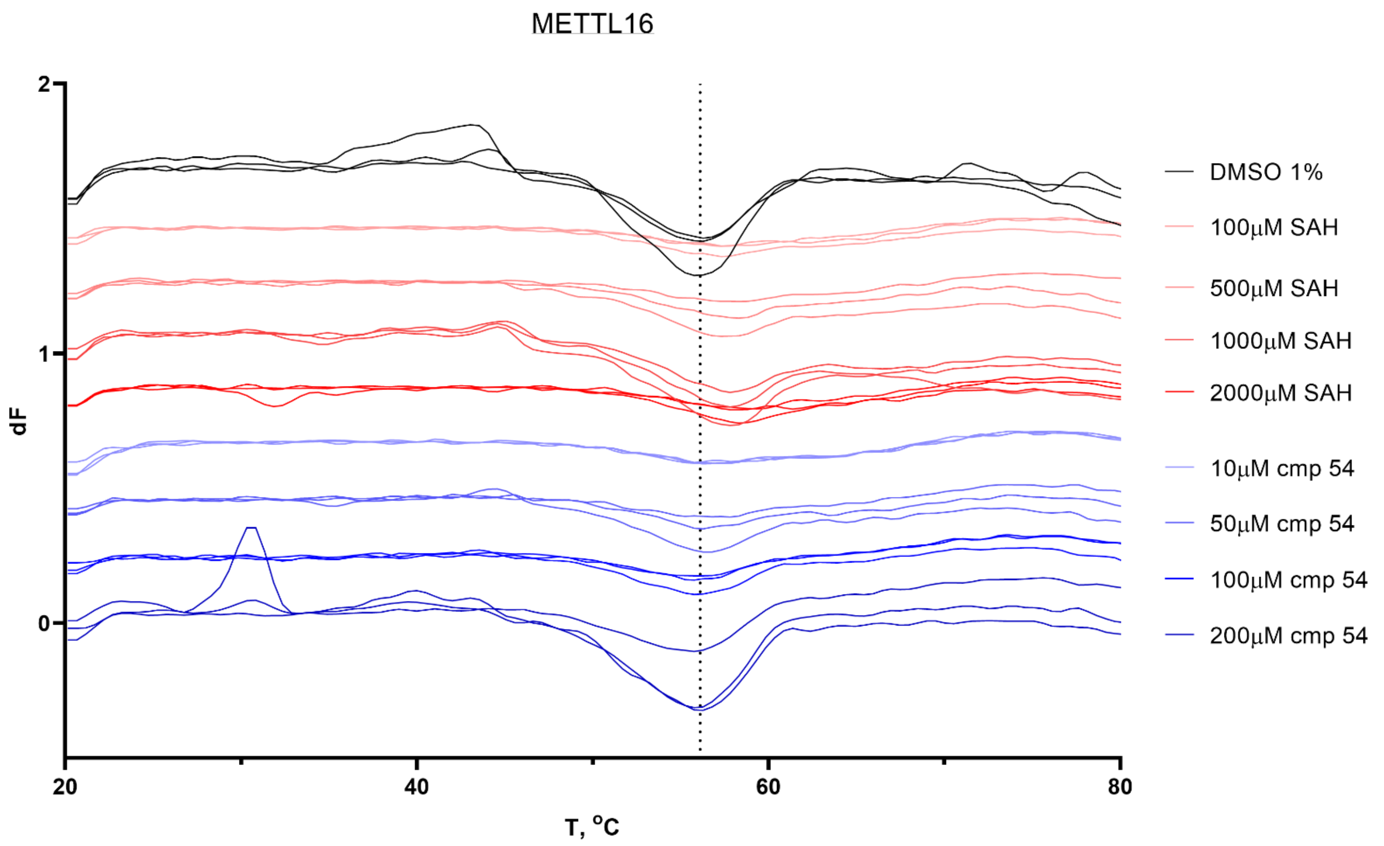

Figure S6: METTL16 thermal shift assay. Shown are the first derivative of the melting curves of METTL16 in the presence of inhibitor **54** or SAH at different concentrations.

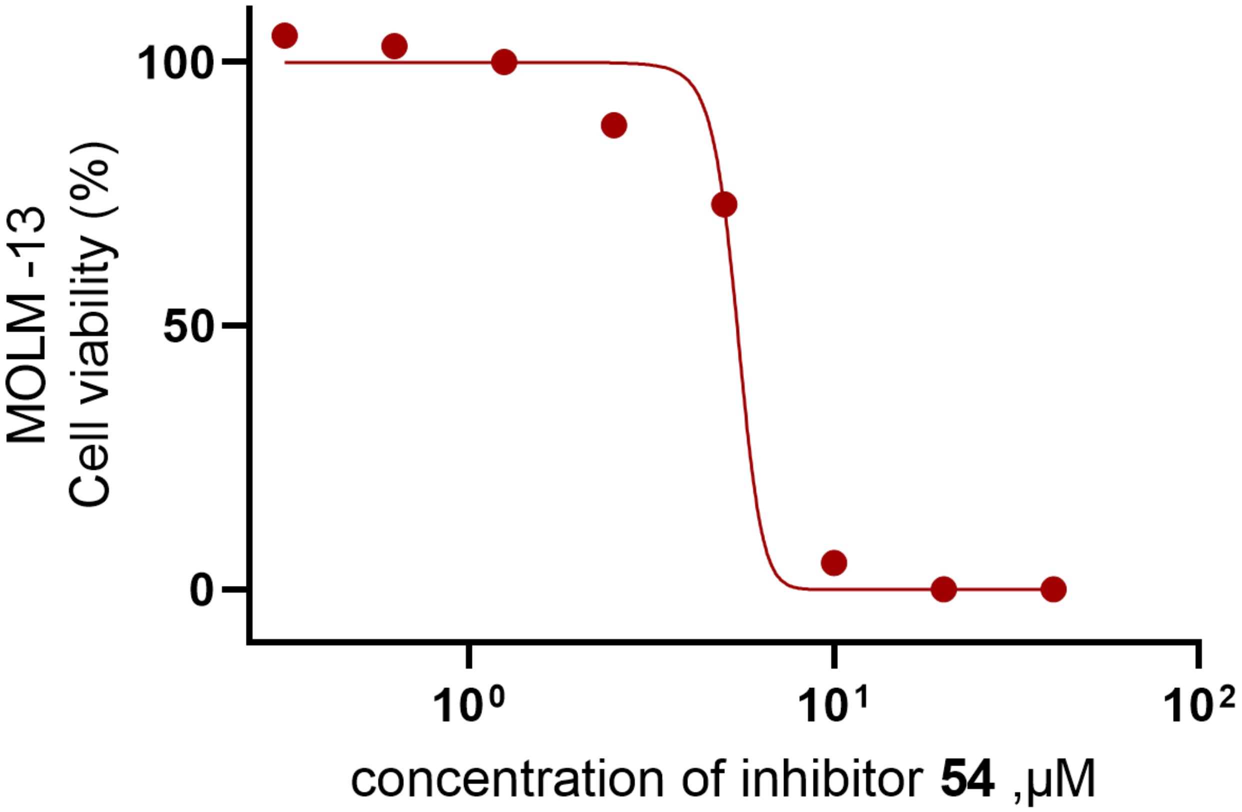

Figure S7: Cell viability curve of MOLM-13 cells upon incubation with inhibitor **54** for 3 days. The fitting results in a GI_{50} value of 6 µM (average of two biological replicates).

Cmp (PDB id)	2D structure	IC_{50} (μM)
**S1**	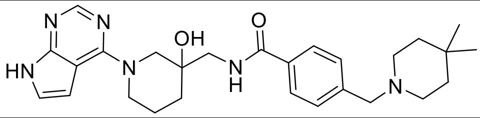	9.8
**S2** (7OED)	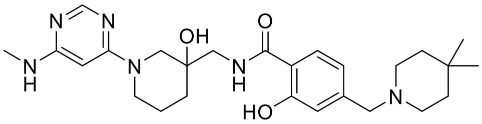	2.6
**S3**	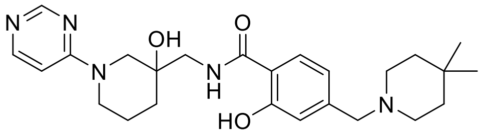	57
**S4**	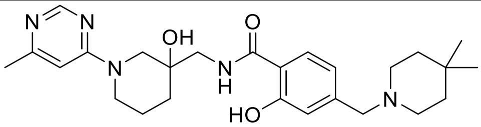	51
**S5** (7OEJ)	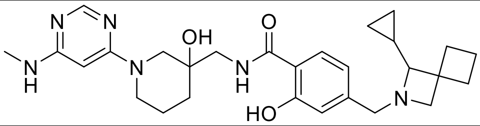	4.9
**S6** (7OQO)	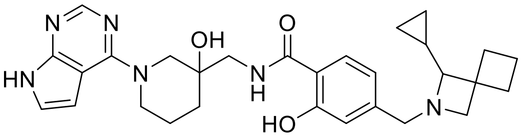	0.74
**S7** (7OQP)	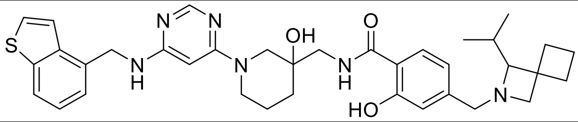	0.18
**S8**	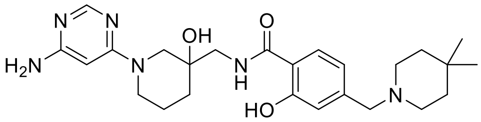	4.1

Table S1: Combination of structural features. The IC_{50} values were determined for the racemic mixtures by an assay based on homogeneous time-resolved fluorescence (HTRF).

Cmp	2D structure	IC50 (µM) Racemate	IC50 (µM) *R*	IC50 (µM) *S*	*S*/*R* Ratio
**S1**	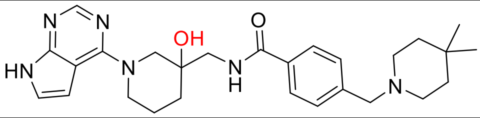	9.8	4.5	26	5.8
**16**	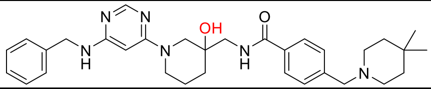	1.7	1.2	7.1	5.9
**S2**	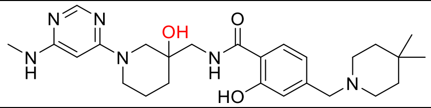	2.6	2.9 (7OED)	9.7 (7OEE)	3.3
(UZH1) **43**	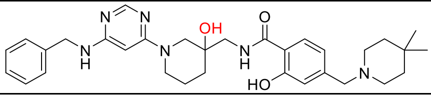	0.48	0.29 (7ACD, UZH1a)	27 (UZH1b)	93.1
**29**	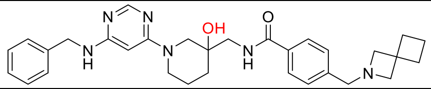	1.6	0.53 (7NI8)	1.3 (7OEG)	2.5
**31**	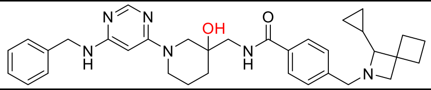	0.38	0.35 (7NIA)	1.3 (7OEH)	3.7
**46**	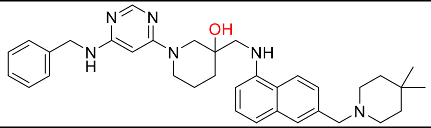	0.36	0.2 (7NID)	12	60.0

Table S2: Racemate, *R* and *S* isomer IC_{50} comparison. The IC_{50} values were determined by the HTRF assay.

State	System (compound ID)	Simulation length of each run (ns)	Number of runs	PDB ID
Apo METTL3/14	Apo	500	5	5K7M
Holo METTL3/14	**1**	100	3	7NHG
	**2**	100	3	7NHJ
	**43***R*	100	5	7ACD
	**43***S*	100	5	7ACD (Protein) and 7OEE (Ligand)
	**16***R*	100	5	7NHV
	**16***S*	100	5	7ACD (Protein) and 7OEE (Ligand)

Table S3. Molecular dynamics (MD) simulations of apo METTL3/14, and in the complex with six inhibitors.

Compound ID	$\Delta E(R_{local})$	$\Delta E(R_{global})$	$\Delta E\ (S_{local})$	$\Delta E(S_{global})$	$\Delta\Delta E(RS_{local})$	$\Delta\Delta E(RS_{global})$
**16**	3.8	11.8	5.6	15.4	1.8	3.6
**S2**	2.8	8.5	5.3	10.0	2.4	1.5
**46**	3.1	13.3	6.6	16.2	3.5	2.8
**43**	3.7	12.6	4.6	13.7	0.9	1.1

Table S4. Molecular dynamics (MD) simulations of inhibitors in the unbound state.

Conformer (Ligand) strain energies of *R* and *S* configurations of METTL3 inhibitors. The ligand strain energy was calculated by the Freeform module implemented in the OpenEye package. All values are in kcal/mol.

$\Delta E(R_{local})$: The local conformer strain energy of the *R* configuration.

$\Delta E(R_{global})$: The global conformer strain energy of the *R* configuration.

$\Delta E\ (S_{local})$: The local conformer strain energy of the *S* configuration.

$\Delta E(S_{global})$: The global conformer strain energy of the *S* configuration.

$\Delta\Delta E(RS_{local}) = \Delta E\ (S_{local}) - \Delta E(R_{local})$

$\Delta\Delta E(RS_{global}) = \Delta E(S_{global}) - \Delta E(R_{global})$

NMR traces of compound **54**

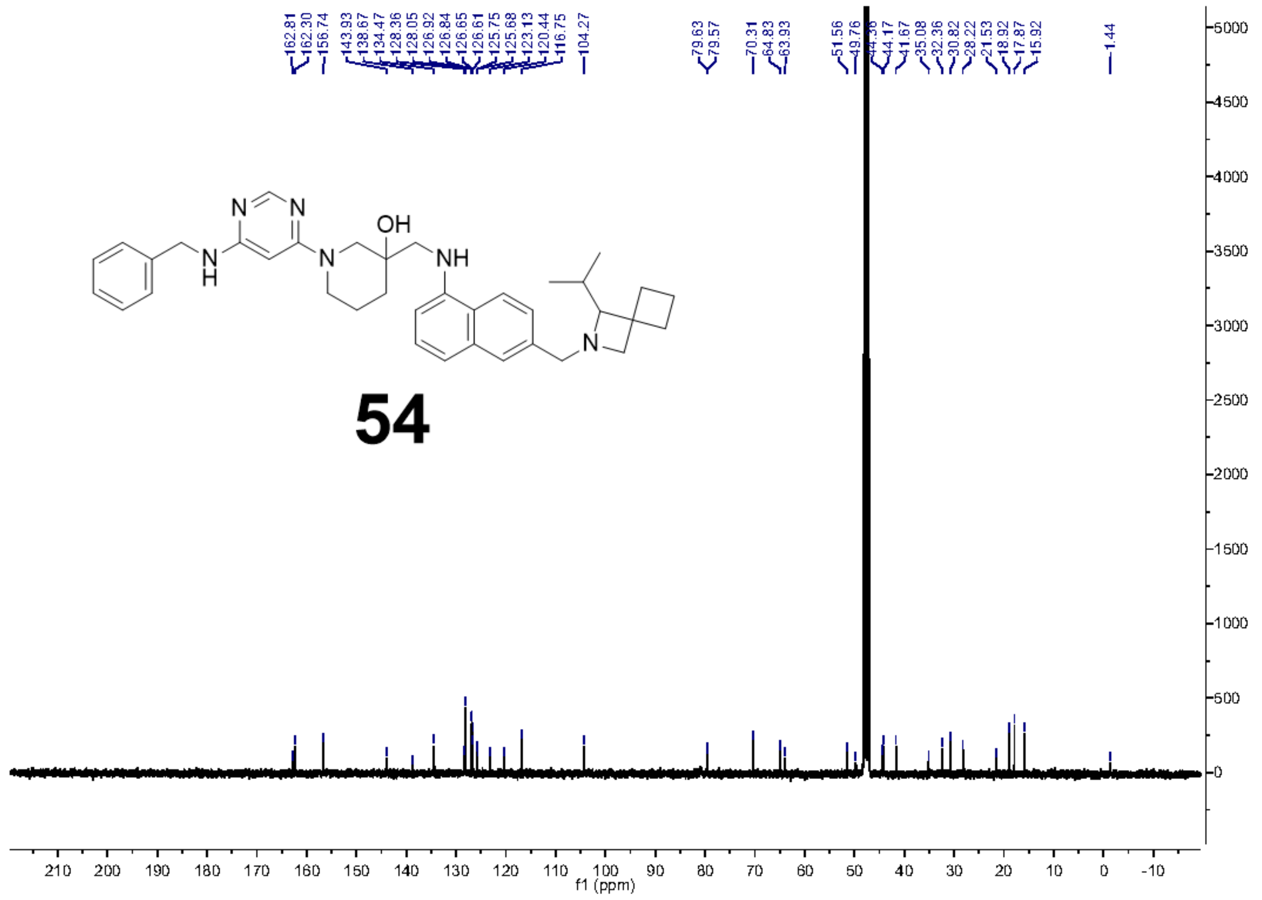

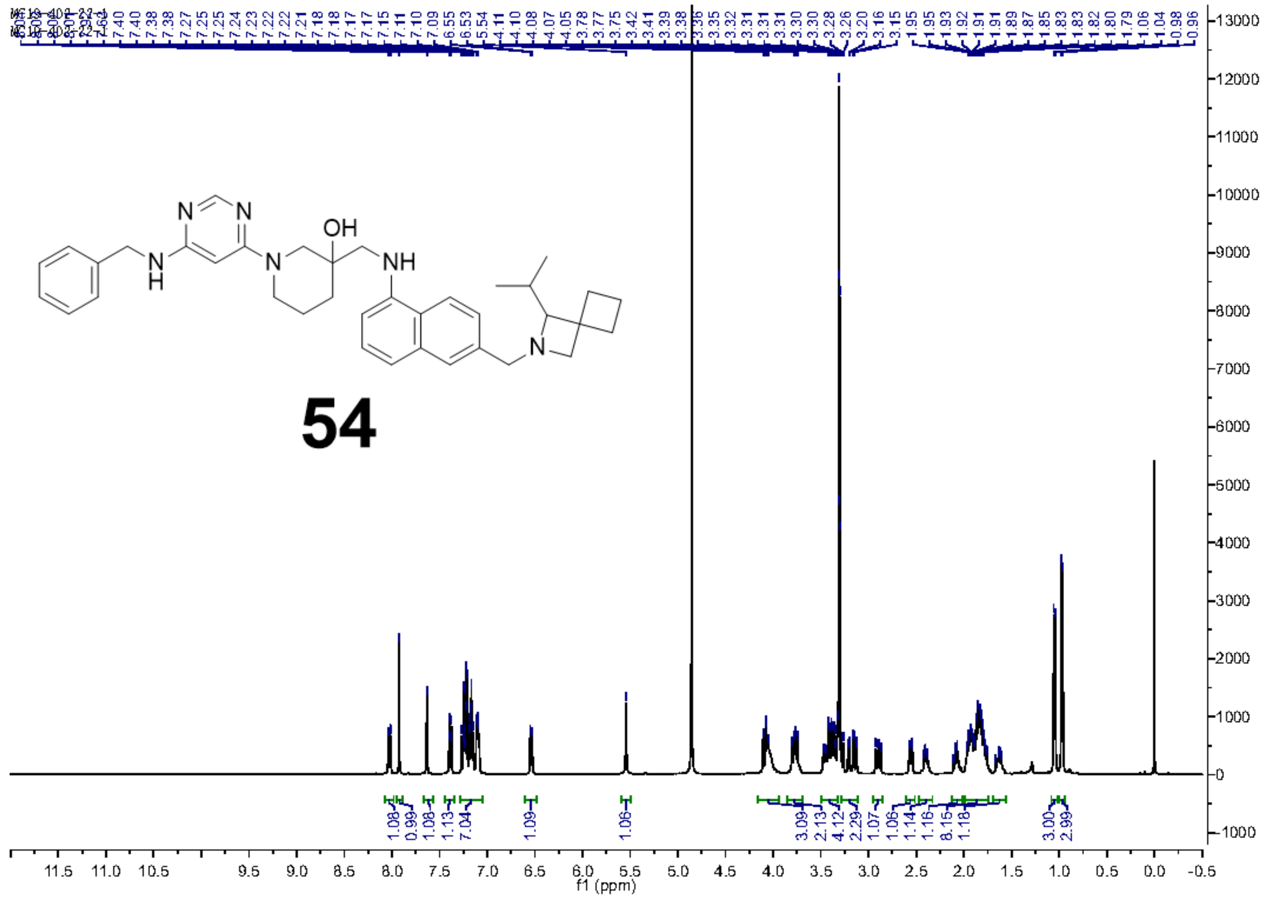

LCMS (ESI): m/z 590 $(M+H)^+$

1H NMR (400 MHz, CD3OD) δ 8.02 (dd, *J* = 8.8, 3.2 Hz, 1H), 7.92 (s, 1H), 7.63 (s, 1H), 7.39 (dd, *J* = 8.8, 1.6 Hz, 1H), 7.30 - 7.02 (m, 7H), 6.54 (d, *J* = 7.6 Hz, 1H), 5.54 (s, 1H), 4.15 - 3.94 (m, 3H), 3.86 - 3.66 (m, 2H), 3.49 - 3.32 (m, 4H), 3.28 - 3.10 (m, 2H), 2.95 - 2.83 (m, 1H), 2.61 - 2.51 (m, 1H), 2.46 - 2.32 (m, 1H), 2.14 - 2.00 (m, 1H), 2.00 - 1.70 (m, 8H), 1.69 - 1.53 (m, 1H), 1.05 (d, *J* = 6.4 Hz, 3H), 0.97 (d, *J* = 6.4 Hz, 3H).

HPLC traces of compound **54**

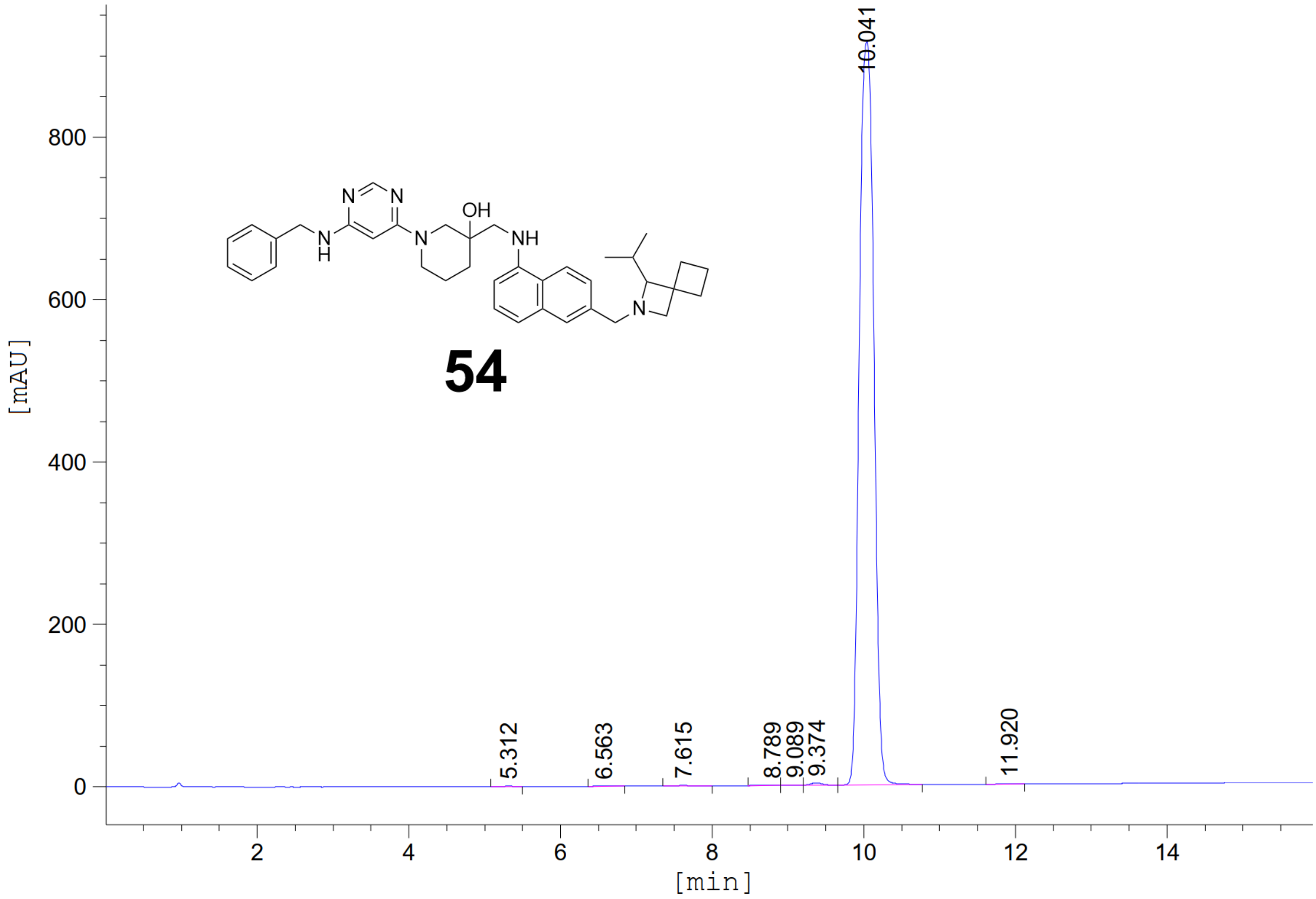

Peak #	RetTime [min]	Type	Width [min]	Area [mAU*s]	Height [mAU]	Area %
1	5.312	VB	0.1013	4.15961	6.44500e-1	0.0341
2	6.563	BB	0.1582	4.23659	3.73996e-1	0.0347
3	7.615	BB	0.1826	8.08794	6.26759e-1	0.0663
4	8.789	BV	0.1895	6.34993	4.64454e-1	0.0520
5	9.089	VV	0.1776	7.92417	6.01896e-1	0.0649
6	9.374	VV	0.1685	32.02310	2.90687	0.2623
7	10.041	VB	0.2167	1.21401e4	915.53906	99.4425
8	11.920	MM R	0.3422	5.27462	2.56878e-1	0.0432

Totals : 1.22082e4 921.41442
